# ZBTB28 induces autophagy by regulation of FIP200 and Bcl-XL facilitating cervical cancer cell apoptosis

**DOI:** 10.1186/s13046-021-01948-0

**Published:** 2021-04-30

**Authors:** Li Li, Yijia Gong, Ke Xu, Weihong Chen, Jiuyi Xia, Zhaobo Cheng, Lili Li, Renjie Yu, Junhao Mu, Xin Le, Qin Xiang, Weiyan Peng, Junying Tang, Tingxiu Xiang

**Affiliations:** 1 Department of Gynecology, The First Affiliated Hospital of Chongqing Medical University, Chongqing, China; 2 Chongqing Key Laboratory of Molecular Oncology and Epigenetics, The First Affiliated Hospital of Chongqing Medical University, No. 1 Youyi Road, Chongqing, 400016 Yuzhong District China; 3 Cancer Epigenetics Laboratory, Department of Clinical Oncology, State Key Laboratory of Translational Oncology, Sir YK Pao Center for Cancer and Li Ka Shing Institute of Health Sciences, The Chinese University of Hong Kong, Hong Kong, China

**Keywords:** ZBTB28, Cervical cancer, Autophagy, Apoptosis, BECN1, FIP200

## Abstract

**Background:**

Among the common preventable cancers of women, cervical cancer has the highest morbidity. It is curable if detected at an early stage. However, reliable diagnostic and prognostic markers, which relate to physiologic and pathologic regulation of cervical cancer, are not available. In this study, one such potential marker, ZBTB28, was evaluated for its potential usefulness in cervical cancer assessment.

**Methods:**

Public database analysis, reverse-transcription polymerase chain reaction (PCR), and methylation-specific PCR were employed to analyze ZBTB28 expression and promoter methylation. The importance of ZBTB28 in cervical cancer cells was assessed by cellular and molecular analysis in vitro and in vivo.

**Results:**

This study assessed the anti-tumor effects of the transcription factor, ZBTB28, which is often silenced in cervical cancer due to CpG methylation of its promoter. We found *ZBTB28* to directly affect cervical cancer cell proliferation, apoptosis, autophagy, and tumorigenesis. Also, it increased cancer cell chemosensitivity to Paclitaxel, Cisplatin, and 5-fluorouracil. Ectopic ZBTB28 expression inhibited the growth of cervical cancer xenografts in nude mice. Furthermore, electron microscopy demonstrated ZBTB28 to induce autophagosomes in cervical cancer cells. ZBTB28 induced cellular autophagy by the degradation of *Bcl-XL*, reduction of the Bcl-XL-BECN1 complex, and by interaction with the autophagy-related gene *FIP200*. ZBTB28-induced autophagy of cervical cancer cells was shown to mediate cellular apoptosis through the regulation of FIP200.

**Conclusion:**

These findings identify ZBTB28 as a tumor suppressor gene that can induce autophagy-related apoptosis in cervical cancer cells. As such, ZBTB28 may be a target for the treatment of uterine-cervical carcinoma. Further, ZBTB28 promoter methylation analysis may offer a new objective strategy for cervical cancer screening.

**Supplementary Information:**

The online version contains supplementary material available at 10.1186/s13046-021-01948-0.

## Background

Cervical cancer ranks fourth in the global incidence of female tumors. Despite continuous improvements in traditional treatment methods, cervical cancer causes more than 300, 000 deaths worldwide each year [1]. It is well known that the development of cervical cancer is a multi-step carcinogenic process, which is a consequence of the activation of multiple oncogenes and the inactivation of tumor suppressor genes [2]. Many cases of cervical cancer are diagnosed at advanced and incurable stages, when traditional medicines and surgical treatments are not satisfactory [3]. To improve outcomes for cervical cancer patients, it is essential to identify effective early prognostic biomarkers.

ZBTB28 (also known as BCL6B, ZNF62, and BAZF) is a recently discovered tumor suppressor gene, which belongs to the POK/ZBTB family and was originally discovered by cloning the homologous BCL6 gene [4, 5]. ZBTB28 has been found in a variety of human tumors and is down-regulated by promoter methylation. This tumor suppressor gene produces anti-cancer effects by inhibition of cellular proliferation, viability, invasion, and migration, as well as the promotion of apoptosis [6, 7]. Remarkably, we show for the first time, by transmission electron microscopy, that overexpression of ZBTB28 induces autophagosomes in the cervical cancer cell lines CaSki and HeLa.

Macroautophagy (hereafter autophagy) is an evolutionarily conserved cellular catabolic process that delivers intracellular components such as cytoplasmic macromolecules and organelles to lysosomes for degradation [8, 9]. It plays an important role in human cancer, infectious disease, and neurodegenerative disorders. It is also involved in various physiological pathways including the process of cellular apoptosis [10–12]. Canonical autophagy is regulated by autophagy-related genes including BECN1, ATG5, and ATG7 that play key roles during the different stages of autophagy [13, 14]. More recently, an alternative autophagy pathway, independent of BECN1/ATG5/ATG7, has been identified [15, 16]. For example, FIP200 was shown to have a crucial function in the regulation of mammalian cells autophagy via the FIP200-ULK1/2-ATG13-ATG101 complex, which can initiate both the canonical and the alternative pathway of autophagy [17]. Numerous studies have indicated that autophagy might serve as a double-edged sword during tumor development. How autophagy acts as both a protector and executioner of cancer cell death is unknown. For example, autophagy can increase tumor cell tolerance to stressors, promoting cancer cell survival in unfavorable environments. Autophagy can also inhibit tumor occurrence and metastasis, promoting tumor cell death through apoptotic pathways [18, 19]. Anti-apoptotic proteins such as Bcl2 and Bcl-XL inhibit autophagy by interacting with BECN1, an autophagy-inducing protein that contains a BH3 domain [20]. This observation suggests the necessity for a context-specific definition of autophagy function in tumor development. An increasing number of studies have demonstrated the signaling pathways of autophagy and apoptosis to be inextricably linked and synergistic, while significantly different with regard to morphology and metabolic pathways [21–24]. For example, autophagy and apoptosis supplement each other to suppress gastric cancer [21], breast cancer [25], leukemia [23], and other tumors. However, an anti-cancer role for ZBTB28 in cervical cancer or even in the induction of autophagy and/or apoptosis is unknown.

In this study, we characterized suppressive functions for ZBTB28 relevant to the occurrence of cervical cancer. We demonstrated that re-expression of ZBTB28 inhibited cervical cancer cell growth and motility, as well as promoted autophagy-related apoptosis. In vivo, ZBTB28 decreased cervical carcinoma xenograft growth and inhibited experimental lung metastasis. Moreover, ZBTB28 increased the sensitivity of cells for the anti-cancer drugs Paclitaxel, Cisplatin, and 5-fluorouracil (5-FU). Further, mechanistic studies showed that stimulation of FIP200 and reduction of Bcl-XL expression, which was crucial to ZBTB28-induced autophagy and apoptosis. These results demonstrate ZBTB28 to play a crucial role in the regulation of the biological function of cervical cancer cells, and as such may serve as a potential candidate for diagnosis and therapy of cervical cancer patients.

## Materials and methods

### Cell lines, tumor samples and normal tissues

Cell lines (Hela, CaSki, HEK293T) were obtained from the American Type Culture Collection (ATCC, Manassas, VA, USA) and collaborators. Cancer cell lines (Hela, CaSki) were cultured in RPMI 1640 medium (Gibco-BRL, Karlsruhe, Germany) with 10% fetal bovine serum (FBS), and 293 T cells were grown in high glucose DMEM (Gibco-BRL, Karlsruhe, Germany) supplemented with 10% FBS. All cells were cultured at 37 °C in a humidified atmosphere containing 5% CO_2_.

All tissue samples used were acquired from the First Affiliated Hospital of Chongqing Medical University, including primary tumor and paired surgical margin tissues as well as normal tissues. In order to make sure that the percentage of tumor cells was over 70%, tissue samples were pathologically and histologically examined with collection of clinical and pathological data followed. This study was conducted according to provisions of the Helsinki Declaration in 1975 and authorized by the Institutional Ethics Committees of the First Affiliated Hospital of Chongqing Medical University (Approval notice: No. 20180305).

### Construction of plasmids and transfection

To construct the ZBTB28 expression plasmid, the *Homo sapiens ZBTB28* full-length gene with a HA-tag was cloned into the pcDNA-3.1(+) framework plasmid. The recombinant plasmids were transformed into *E. coli* DH5a cells (CB101, TIANGEN) and then sequenced. Cells were transfected with plasmid DNA using Lipofectamine 2000 reagent (Invitrogen) according to the manufacturer’s instructions. Stably transfected pcDNA-3.1 and pcDNA-ZBTB28 cells were acquired by G418 selection (600 μg/ml for CaSki, 800 μg/ml for HeLa).

### RNA isolation, reverse transcriptase-PCR and real-time PCR

Learning from the manufacturer’s instructions, total RNA was isolated from cell lines and clinical samples using TRIzol Reagent® (Molecular Research Center, Cincinnati, OH, USA). Twenty microliter cDNA was synthesized from 1 μg RNA by Reverse transcriptase-polymerase chain reaction (RT-PCR). Real-time PCR was performed by using Go-Taq (Promega, Madison, WI, USA) under the following conditions: initial denaturation at 95 °C for 2 min, followed by 32 cycles (95 °C for 30 s, 55 °C for 30 s and 72 °C for 30 s) of amplification, and final extension at 72 °C for 3 min. β-actin was amplified as a control and with 23 cycles. Semi-quantitative RT-PCR, with β-actin as a control, was put into practicing with Go-Taq DNA polymerase (Promega) and performed by using a final volume of 10 μL reaction mixture that contained 2 μL cDNA. The amplified products were electrophoretic in 2% agarose gel to detect the target bands. In the case of SYBR Green (Thermo Fisher), Real-time quantitative PCR had performed. With the action of 7500 Real-Time PCR System (Applied Biosystems, Foster City, CA, USA), all analyses were performed. The primer sequences are shown in Table S2 and Table S3.

### 5-Aza-2′-deoxycytidine (5-Aza) treatment

CaSki and Hela cell lines were treated with 10 μmol/L 5-Aza (Sigma-Aldrich, Steinheim, Germany), a demethylation agent, for 4 days. Then cells were harvested for qRT-PCR analysis.

### Bisulfite treatment, methylation specific PCR (MSP) and quantitative methylation specific PCR (qMSP)

To evaluate ZBTB28 methylation status, bisulfite modification of DNA was performed [26]. By using AmpliTaq-Gold DNA Polymerase (Applied Biosystems), MSP was then exercised with methylation-specific primers to specifically recognize unmethylated or methylated *ZBTB28* gene sequences. Products were electrophoresis with 2% agarose gels (MBI Fermentas, Vilnius, Lithuania) and were recorded on a gel imaging system (Bio-RAD Gel Doc XR+, CA). The primers of qMSP were mentioned before [7].

### Tumor xenograft model and metastasis models in nude mice

All animal experiments in this study were conducted in compliance with animal protocols approved by the Experimental Animal Center of Chongqing Medical University, and were approved by the institutional ethics committee of the First Affiliated Hospital of Chongqing Medical University, China. Six BALB/c nude mice (aged 4–6 weeks, weighing 18–22 g) were purchased from Beijing Vital River Laboratory Animal Technology Co. Ltd. Stable ZBTB28- expression Hela cells or control cells (5 × 10^6^ cells were resuspended in 100 ul PBS) were injected into subcutaneous tissues on both sides of the back of 6 nude mice. The primary tumor size was measured on the fourth day after injection. The second measurement was performed on the eighth day. And then measurements had been taken every 2 days until the 18th day after injection. The longest and the shortest diameters of tumors were measured by using vernier calipers, and tumors’ volume (mm^3^) were calculated as follows: volume = length × width^2^ × 0. 52. All nude mice were euthanized the 19th day after injection, and their tumors were collected and weight. Some of the collected tumor tissues were used for embedding sections, and some were frozen in − 80 °C refrigerator for subsequent experiments. As previously described [27], female BALB/c nude mice (5–6 weeks) were used in metastasis assays. Two weeks after tail vein injection, the mice were sacrificed and checked for lung metastasis using standard histological examination (Approval notice: No. 20180226).

### Flow cytometry (FCM)

Cell cycle arrest and apoptosis were assayed by flow cytometry (FCM). For cell cycle analysis, stably transfected cells were harvested with trypsin, then washed twice with PBS, suspended with ice-cold 75% ethanol and fixed overnight. The next day, cells added with RNA enzyme were incubated at 37 °C for 30 min, and stained with propidium iodide (PI) for 30 min in dark to assay for cell cycle distribution. In order to analyze apoptosis, transiently transfected cells were double staining with annexin V-fluorescein isothiocyanate and PI. A Cell Quest kit (BD Biosciences, CA, USA) was employed to assess FCM results.

### Cell proliferation (CCK8)

CaSki and Hela cells were cultured in 96-well plates at a density of 3000 cells/well after transfection with *ZBTB28*-expressing or control (pcDNA3.1) plasmids. The absorbance of cells in each well at 450 nm was measured with CCK8 kit at 0 h, 24 h, 48 h, 72 h, and the mean value of each well was obtained after testing nine times.

For the half maximal inhibitory concentration (IC50) determination, cells were treated with progressively increased concentrations of drugs for 72 h. Cisplatin (HY-17394), Paclitaxel (HY-B0015) and 5-FU (HY-90006) were purchased from MedChemExpress. The value of IC50 was calculated by nonlinear regression analysis with software program GraphPad Prism version 5.

### Colony formation assays

Stably transfected pcDNA-3.1 and pcDNA-ZBTB28 cells (CaSki and Hela) were plated in six-well plates (200 cells/well). After 12 days, cells were fixed with 4% Paraformaldehyde (PFA) for 30 min and stained with gentian violet (Beyotime Institute of Biotechnology, Shanghai, China). Photographed with a phase contrast microscope (Leica DMI4000B, Milton Keynes, Buckinghamshire, UK), colonies with more than 50 cells were manually counted with Photoshop software.

### Transwell® assays for cell migration and invasion

For Transwell® assays, the chambers (8 μm pore size, BD Sciences, Bedford, MA) with or without Matrigel (100 μg/ml, BD Biosciences, San Jose, CA) were used to assess cell migration and invasion capacities. For migration assay, collected cells were washed twice in serum-free medium and added to the upper compartment (3 × 10^4^ cells). The lower chamber contained 700 μl RPMI 1640 medium containing 20% fetal bovine serum (FBS). For invasion assay, the matrix gel was precoated on the upper compartment. After incubation for 48 h, cells were fixed with 4% paraformaldehyde for 30 min and stained for 30 min with crystal violet. Non-migratory cells on the upper side of the chamber were wiped away gently. Photographed with a phase-contrast microscope (Leica DMI4000B) after fixation and staining, migrated cells were counted. Four fields of view were randomly selected for counting.

### Immunofluorescence and Western blot

Immunofluorescence was performed as Qadeer ZA et al. described [28]. Cells grew on round coverslips were incubated with HA-tag (#3724, Cell Signaling Technology), anti-Vimentin (sc-6260, Santa Cruz), and anti-E-cadherin (#14472, Cell Signaling Technology) antibodies and secondary antibodies conjugated with Alexa Fluor 488 and 594. Nuclei were counterstained with DAPI (C1006, Beyotime Biotechnology, Haimen, China). Images were acquired on a fluorescence microscope (Olympus, Tokyo, Japan) using 40 × objective.

Western blot was performed to determine the levels of the autophagic proteins p62 (#8025, Cell Signaling Technology), LC3 (ab128025, abcam), apoptosis proteins Cleaved Caspase8 (WL00659, Wanleibio, China), PARP and Cleaved PARP (WL01932, Wanleibio, China), Caspase3 and Cleaved Caspase3 (WL02117, Wanleibio, China), EMT proteins Occludin (TA306787, OriGene Technologies), Vimentin (sc-6260, Santa Cruz), N-cadherin (610,921, BD Biosciences) and other primary antibodies HA-tag (#3724, Cell Signaling Technology), Bcl-XL (sc-8392, Santa Cruz), BECN1 (sc-48,341, Santa Cruz), FIP200 (PA5–69698, Thermofisher). The β-actin (sc-8432, Santa Cruz) which was kept as an internal control in CaSki/HeLa cells during the course of differentiation with or without the presence of CQ or overexpression of ZBTB28. Moreover, EMT related proteins (N-cad and VIM) were also detected. Equal amounts of protein (40 μg) were separated by sodium dodecyl sulfate polyacrylamide gel electrophoresis (SDS-PAGE) and transferred onto polyvinylidene difluoride (PVDF) membranes (Bio-Rad, Hercules, CA, USA). Autophagy inhibitor CQ (HY-17589A) and 3MA (HY-19312) were purchased from MedChemExpress.

The membranes, was firstly blocked with PBST (PBS containing 0. 1% Tween 20) containing with 5% nonfat dry milk at room temperature for 2 h and then washed with PBST for three times, secondly incubated with the primary antibody at 4 °C overnight and then washed with PBST. After that, membranes were incubated with the corresponding secondary antibody (BL001A, BL003A, Biosharp, China) at room temperature for 45 min and then washed with PBST for another 30 min. Protein bands were visualized by Immobilon Western Chemiluminescent HRP Substrate kit (Millipore Corporation, Billerica, MA, USA), and the band detections were within the linear range.

### Co-immunoprecipitation (Co-IP)

Co-IP assay was carried out to investigate the interaction of BECN1 and Bcl-XL protein. Briefly, stable ZBTB28-expressing cells CaSki and HeLa were transfected with Bcl-XL plasmids. Total proteins were extracted with ice-cold low salt lysis buffer, then, the cell lysate was incubated with Bcl-XL (sc-8392, Santa Cruz), BECN1 (sc-48,341, Santa Cruz) or IgG (#2729, Cell Signaling Technology) antibody overnight on a turning wheel at 4 °C. MilliporeSigmaTmPureProteomeTm Protein A/G Mix Magnetic Bead System (#LSKAGAG10, Fisher Scientific, USA) was used to purify co-IP complex, and the low salt lysis buffer was used to wash the beads. Then co-immunoprecipitation complex was analyzed by SDS-PAGE and Western blot.

### ChIP assay

Chromatin immunoprecipitation (ChIP) analysis was performed according to the manual of the SimpleChIP® Enzymatic Chromatin IP Kit (#9003, Cell Signaling Technology). Stably ZBTB28-expressing CaSki/HeLa cells and vector-expressing cells, firstly were washed with RPMI 1640 medium, then were washed with ice-cold PBS devoid of Ca2^+^ and Mg2^+^ and supplemented with a protease inhibitor cocktail (PIC, P8340, Sigma-Aldrich). Afterwards in order to terminate the reaction, DNA and protein were cross-linked with 1% formaldehyde for 10 min at room temperature followed by 0. 125 M glycine for 5 min. Then cells were lysed in lysis buffer (20 mM Tris-HCl, pH 8. 0, 50 mM NaCl, 5 mM CaCl_2_, 1% Triton X-100, PIC) for 10 min and micrococcal nuclease was added for 20 min at 37 °C. In order to acquire 200–900 bp DNA fragments, cells were sonicated on ice of 9 s pulses at 30% amplitude with an ultrasonic cell disruptor (JY88-IIN, Scientz, China) at an interval of 1 min for five sets. The remanent diluted samples, whose lysates were removed 10 μl in advance to serve as an input sample, were incubated with HA-tag antibody (#3274, Cell Signaling Technology) overnight at 4 °C, followed by capture with protein A/G magnetic beads (#9006, Cell Signaling Technology) for 2 h at 4 °C. Rabbit anti-histone H3 antibody (#4620, Cell Signaling Technology) and normal rabbit IgG (#2729, Cell Signaling Technology) were respectively used as positive and negative controls. As recommended, the complexes were precipitated, washed, and eluted. After DNA-protein cross-linkages were incubated with 6 μl of 5 M NaCl and 2 μl of proteinase K at 65 °C overnight, DNA was washed and purified with Anhydrous ethanol for once, 75% ethanol for 2 times, then drying at 55 °C for 10 min. Fifty microliter of enzyme free water was added for further using in q-PCR analyses. The primer sequences are shown in Table S4.

### Autophagy flux assay

For the autophagy assay, CaSki and Hela cells were imaged by confocal laser scanning microscope, after transfected with eGFP / eGFP-LC3 for 48 h.

### Transmission electron microscopy (TEM)

The cells were stained with uranyl acetate/lead citrate and observed by a Hitachi H-7650 transmission electron microscope (Tokyo, Japan) operated at 100 KV.

### Luciferase reporter assay

Two hundred ninety three T, CaSki and HeLa cells were seeded in 24-well plates and grown to 50–60% confluence. Then the cells were co-transfected with pGL3-gene, HA-ZBTB28, and monitor plasmid pRT-LK (80:1 ratio). After 48 h, the cells were lysed in 100 μl lysis buffer and the Firefly and Renilla luciferase activities were detected by the Dual-Luciferase Reporter System (Promega) according to the manufacturer’s instructions. After the luciferase activity of the tested sample had been normalized, data are represented as the fold induction to that of the corresponding control sample.

### Spheroid forming assay

Spheroid culture was performed as previously described [7]. Briefly, 2000 cells were seeded onto 6-well plate, after 2 weeks, the spheroids were examined by using microscope (Olympus, Tokyo, Japan). The number of spheroids was calculated using captured images. Spheroid cells larger than 50 μm were considered as formed spheroids. The efficiency of spheroid formation is the percentage of spheroid cells in live cells seeded.

### Acridine orange (AO)/ethidium bromide (EB) staining

AO/EB double staining was used to detect apoptosis (A simple technique for quantifying apoptosis in 96-well plates). Briefly, 5000 cells/well were seeded in a 24-well plate. After 24 h, gently washing with 1× PBS, then the cells were stained with acridine orange (100 μg/ml) and ethidium bromide (100 μg/ml) mixed solutions for 1 min (kit). After staining the cells were washed with 1× PBS, then observed under a microscope (Olympus, Tokyo, Japan) and recorded.

### EdU (5-ethynyl-2′-deoxyuridine) incorporation assay in vitro

EdU detection was performed using the BeyoClickTM EdU Cell Proliferation Kit with Alexa Fluor 488 (Beyotime Biotechnology, Haimen, China), according to manufacturer’s instructions. In brief, cervical cancer cells were incubated with 10 μM EdU for 2 h at 37 °C. Cells were then fixed with 4% paraformaldehyde and permeabilized with 0. 3% Triton X-100 for 15 min at room temperature. Next, the fixatives were removed, and the cells were washed with PBS containing 3% BSA (4240GR005, BioFroxx). Last, cells were incubated from light in Click Additive Solution for 30 min then stained nucleus with Hoechst. The pictures of EdU detection samples were then acquired under microscope (Olympus, Tokyo, Japan) and photographed. The cell proliferation was further analyzed by counting the ratio of EdU incorporated cells to the total number of cells.

### Immunohistochemistry (IHC)

Briefly, paraffin sections were dewaxed, washed twice in absolute ethanol, follow the instructions (ZSGB-BIO, SP9000) for hydration and antigen retrieval. Next, incubated slides with reagent 1 for 10 min at room temperature to inactivate endogenous peroxidase, and followed by three times washes in PBS for 3 min. Then sections were incubated in blocking solution (Reagent 2) for 15 min, continued by overnight incubation at 4 °C with PCNA (#13110, Cell Signaling Technology), HA-tag antibody in immunohistochemical wet box. Next day, sections were three washes with PBS, then incubated with reagent 3 at 37 °C for 30 min in wet box, washed again in PBS, and incubated with reagent 4 at 37 °C for 30 min. After three times washed with PBS, immunolabeling was performed with 1x diaminobenzidine (DAB, ZSGB-BIO, ZLI-9018). Finally, the sections were counterstained with hematoxylin (BL702B, Biosharp, China).

### siRNA and transfection

siRNA sequences against human *BECN1* and *FIP200* were purchased from OriGene (OriGene Technologies, Rockville, MD). At a concentration of 10 nM, a pool of three different siRNA duplexes was used to transfections. Transient transfections were performed in 6-well plates using Lipofectamine 2000 (Invitrogen). After transfection 48–72 h, cells were harvested for subsequent assays.

### Statistical analyses

GraphPad Prism 5 was used to conduct the statistical analysis. All datum were presented as the mean ± standard error of the mean (SEM). Statistical analysis was performed by comparing two groups of data in the way of Student t test. The significance of statistical analysis was clarified by *p < 0. 05, **p < 0. 01 and ***p < 0. 001.

## Results

### ZBTB28 is down-regulated by CpG methylation in cervical cancer cells and tissues

**Fig. 1:**
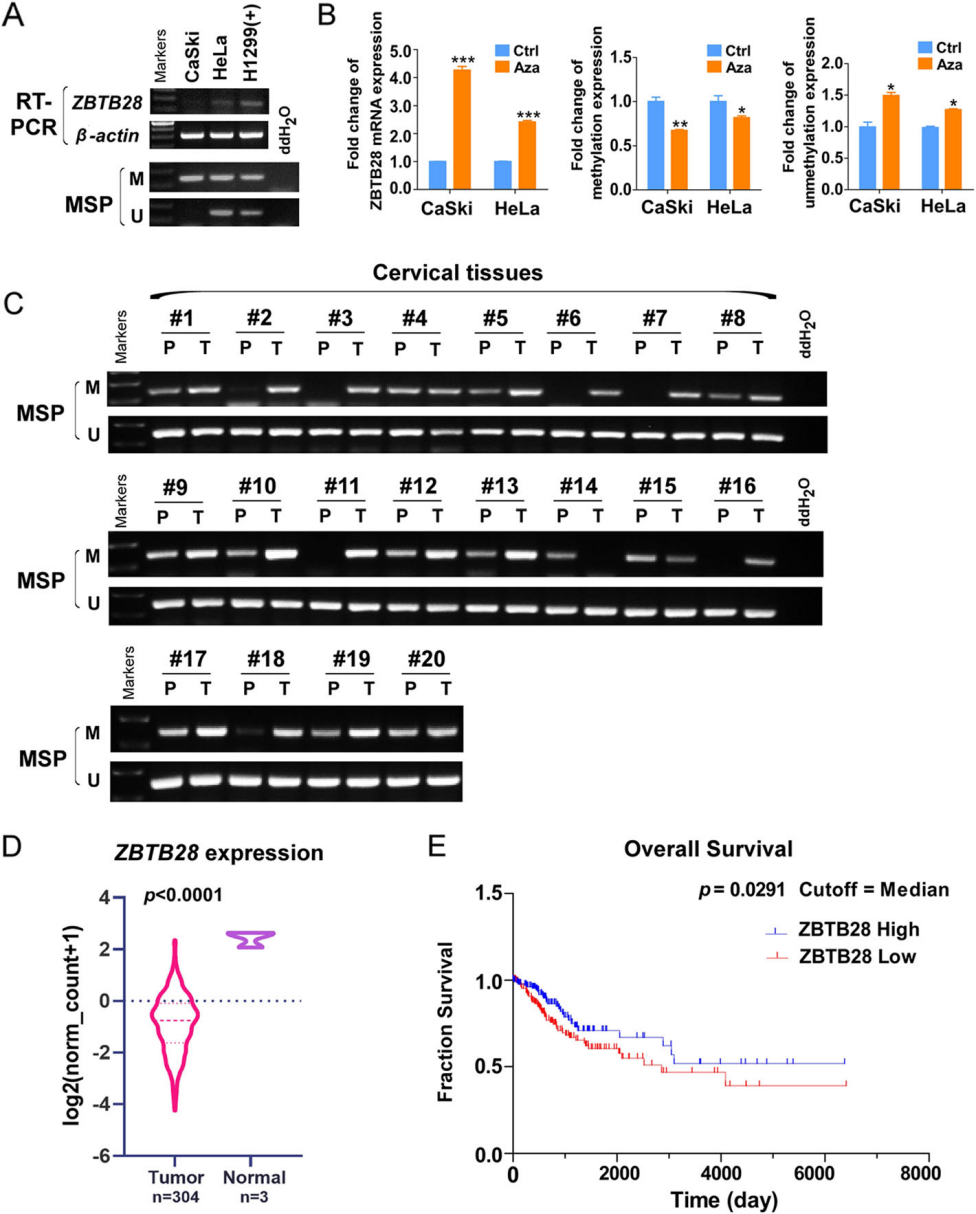
Expression and methylation of ZBTB28 in cervical cancer. **a** *ZBTB28* expression and methylation status in cervical cancer cell lines. H1299 was used as a positive control, and ddH_2_O was used as a negative control. **b** Impact of the 5-Aza treatments on *ZBTB28* expression and methylation status in cervical cancer cell lines. Demethylation was measured by qMSP. **c** *ZBTB28* methylation in primary cervical cancer tissues (*n* = 48) and adjacent non-cancerous tissues (*n* = 20) was measured by MSP. Only representational gel images were shown for illustration. **d** *ZBTB28* expression in cervical cancer and adjacent normal tissues from TCGA. **e** Analyses of the association between *ZBTB28* expression and survival in cervical cancer patients. Data were obtained from the TCGA. M, methylated; U, unmethylated. *p < 0. 05, **p < 0. 01, ***p < 0. 001

We have previously shown that the transcription factor ZBTB28 was a tumor suppressor molecule, which was widely expressed in normal tissues and significantly down-regulated in a variety of cancer types [7]. *ZBTB28* expression was silenced in CaSki and HeLa cells, further methylation-specific PCR (MSP) analysis has revealed that the methylation of *ZBTB28* promoter was correlated with its down-regulation (Fig. 1a). To confirm the role of CpG methylation on the downregulation of *ZBTB28* expression, cervical cancer cells were treated with DNA methylation inhibitor 5-Aza-2-deoxycytidine (5-Aza). As expected, the expression of *ZBTB28* was restored by 5-Aza accompanied with the reduction of methylation level and increase in unmethylated alleles (Fig. 1b). To assess the promoter methylation status, we performed MSP analysis on 48 samples of primary cervical tumors, including 20 paired and 28 tumor tissues. *ZBTB28* methylation was detected in 45/48 (93. 75%) tumors but 13/20 (65%) paired adjacent non-tumor tissue samples (Fig. 1c, Table S1), thus indicating that *ZBTB28* methylation was a common event in cervical cancer. Next, we found that *ZBTB28* was down-regulated among the The Cancer Genome Atlas (TCGA) cervical cancer database (https://tcga-data. nci. nih. gov/tcga/) (Fig. 1d). Survival analysis curve calculated from cervical cancer patients from TCGA illustrated that high expression of *ZBTB28* was positively correlated with longer overall survival (Fig. 1e).

### ZBTB28 suppresses the growth and metastasis of cervical cancer cells

**Fig. 2:**
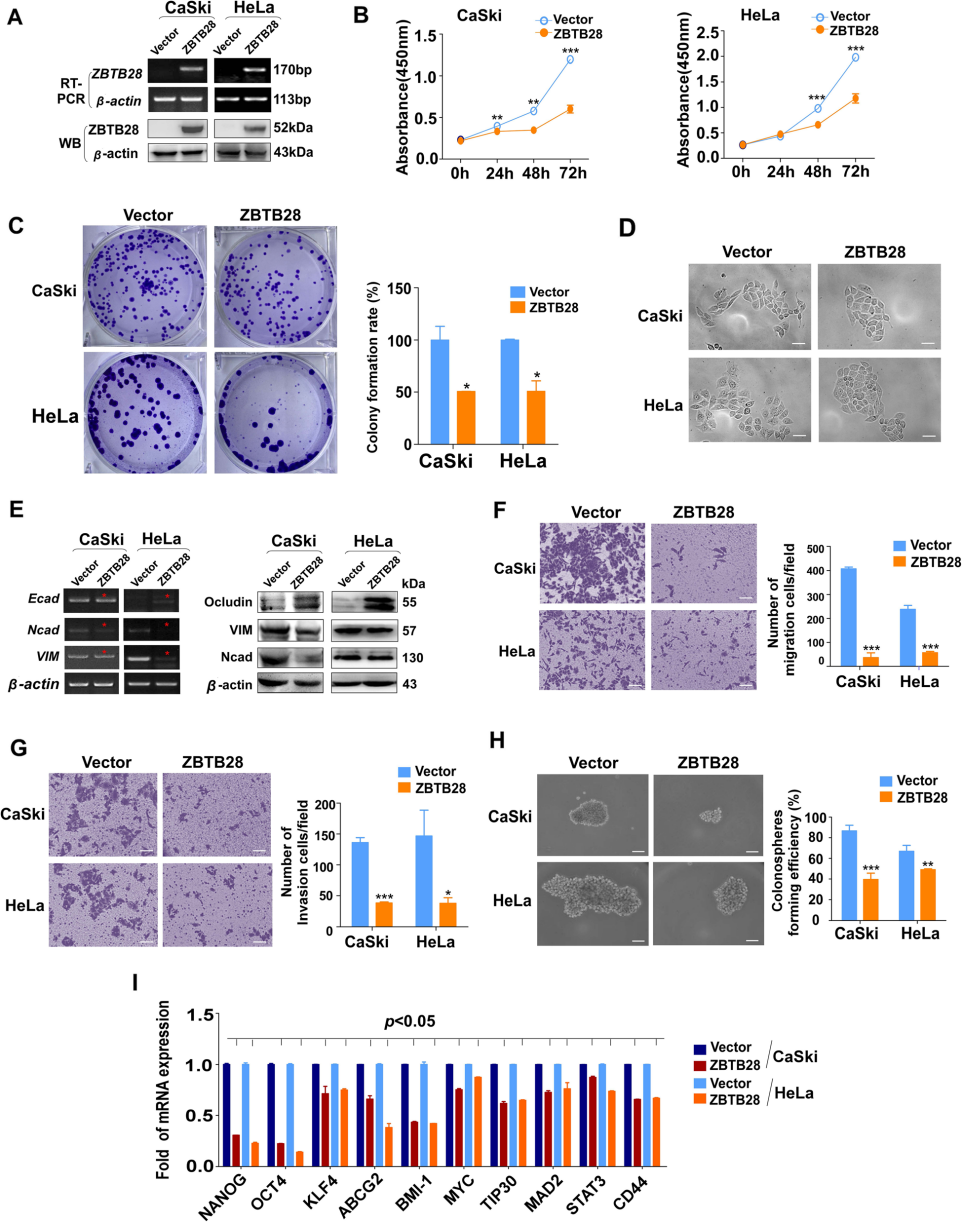
Tumor suppressive functions of ZBTB28 in cervical cancer cells. **a** RT-PCR and western blot analysis confirmed the exogenous expression of ZBTB28 in CaSki and HeLa cells, with β-actin as a control. **b** Ectopic ZBTB28 expression impacts cervical cancer cell growth were analyzed by the CCK8 kit. **c** Re-expression of ZBTB28 suppressed colony formation in CaSki and HeLa cells. **d** Morphological changes of cervical cancer cells that stable transfected with pcDNA-3.1 or pcDNA-ZBTB28 by using phase contrast microscopy. **e** EMT marker were detected by RT-PCR and western blot assay. **f**, **g** Pictures were taken at 48 h after seeding, the relative ratio of migration and invasion cells per field was shown. **h** Representative spheroid-forming cells images were taken at 14 days after seeding. **i** Overexpression of ZBTB28 in cervical cancer cells downregulated the mRNA expression level of stemness-related genes. All statistical data were shown as mean ± SEM. Scale bar: 200 μm; *p < 0. 05, **p < 0. 01, ***p < 0. 001

To analyze the functional of ZBTB28 in cervical cancer, we transfected pcDNA3.1(+) framework plasmid or pcDNA-HA-ZBTB28 plasmid into cervical cancer cell lines CaSki and HeLa which lack endogenous *ZBTB28* expression. Ectopic expression of ZBTB28 was confirmed by Reverse transcription (RT)-PCR and Western blot (Fig. 2a). CCK8 results showed that exogenous ZBTB28 expression inhibited cell viability of both cell lines (Fig. 2b). The inhibitory effect on cell growth was further convinced by colony formation assay, in which ZBTB28 decreased the number and size of colonies in CaSki and HeLa compared with the control cells (Fig. 2c). Interestingly, we observed that the cell morphology was changed after re-expression ZBTB28 in CaSki and HeLa cells, presenting a more adhesive contact pattern while the control cells still showed scattered distribution (Fig. 2d). Therefore, we hypothesized that ZBTB28 might be involved in tumor cell epithelial mesenchymal transition (EMT).

As we all know, EMT is the key process of metastasis and invasion of cancers [29]. At transcription and post-transcription levels, we verified that the epithelial markers E-cadherin and Occludin were up-regulated due to ectopic ZBTB28 while mesenchymal markers N-cadherin and Vimentin were down-regulated in CaSki and Hela cells (Fig. 2e, Fig. S1). Furthermore, we conducted Transwell assay to explore the role of ZBTB28 in metastasis of cervix cancer cells. Within expectation, over-expression of ZBTB28 significantly inhibited the ability of migration and invasion of CaSki and HeLa cells compared with control cells (Fig. 2f, g). Besides cancer cells metastasis and invasion, EMT also closely related to tumor self-renewal and differentiation [29]. The spheroid formation test proved that ZBTB28 decreased the spheroid formation rate compared to controls, indicating that ZBTB28 affects the self-renewal capacity of single cell and the stemness of cervical tumor cells (Fig. 2h). Next, qRT-PCR was used to detect whether ZBTB28 contributed to the regulation of tumor stemness at transcription level. The results illustrated that ZBTB28 down-regulated the expression of *NANOG, OCT4, KLF4, ABCG2, BMI1, MYC, TIP30, MAD2, STAT3* and *CD44* in cervical cancer cells (Fig. 2i). Overall, our study verified that ZBTB28 played an anticancer role through inhibiting the proliferation, growth, migration, invasion and carcinogenicity of cervical cancer cells.

### ZBTB28 induces cell cycle arrest and apoptosis in cervical cancer cells

**Fig. 3:**
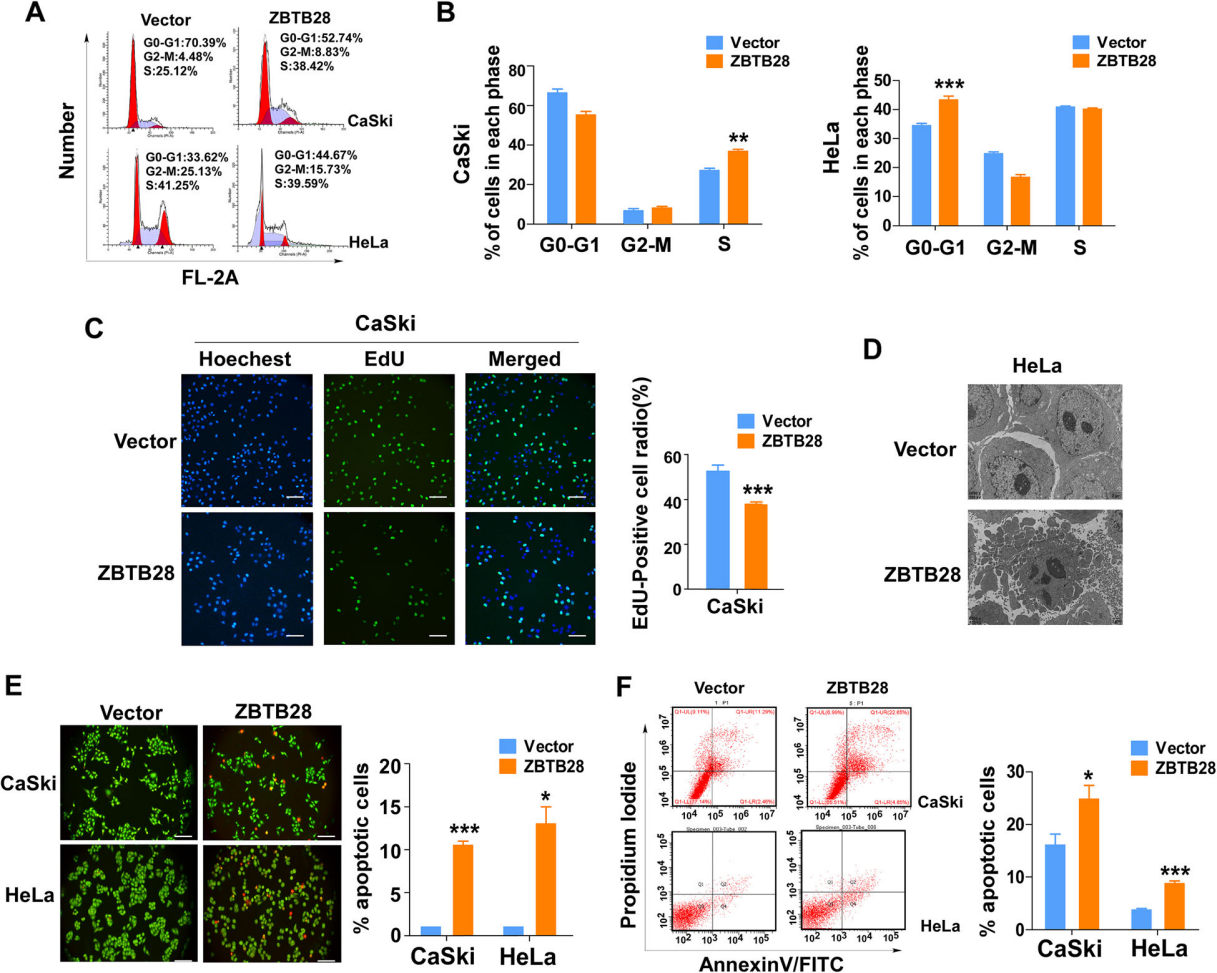
ZBTB28 induced cell cycle arrest and promoted cell apoptosis. **a**, **b** Flow cytometry analysis of cell cycle of CaSki and HeLa cells which was overexpression pcDNA 3.1 or pcDNA-ZBTB28 by PI staining. **c** EdU incorporation in the growing CaSki cells that with/without pcDNA-ZBTB28 were analyzed. **d** Transmission electron micrograph of apoptotic cells: the proportion of apoptotic bodies were increased in ZBTB28-transfected CaSki and HeLa cells, relative to the pcDNA 3.1-transfected control cells (CaSki: 4000x, Scale bar: 5 μm; HeLa: 6000x, Scale bar: 2 μm). **e** Ectopically expressed ZBTB28 induced apoptosis in cervical cancer cells were tested by AO/EB staining assay. **f** Flow cytometry analysis of apoptotic cell population by Annexin V/PI staining. All statistical data were shown as mean ± SEM. *p < 0. 05, **p < 0. 01, ***p < 0. 001

We next explored whether there were other underlying mechanisms about the ability of ZBTB28 in cervical cancer cells for growth inhibition. Cell cycle arrest and/or cell apoptosis often related with low cell viability. Hence, flow cytometry analyses were performed to detect cell cycle and apoptosis. The content of DNA was calculated by PI fluorescence intensity, a fluorescent vital dye that can linearly bind to DNA without sequence preference, which can reveal the cell cycle states [30]. PI staining expounded that, in CaSki cells, cell cycle was arrested in S phase by ZBTB28 re-expression accompanied with a decrease in the proportion of G0/G1 phase while the proportion of G2/M phase remained no statistical difference. In the meanwhile, the proportion of G0/G1 phase cells were increased by over-expression of ZBTB28 in HeLa cells, with slight decrease of G2/M phase cells and S phase (there were no statistical difference) (Fig. 3a, b). To further confirm that the increase of S phase was resulted from cell cycle arrest induced by ZBTB28 rather than DNA replication, we performed EdU incorporation assay in CaSki cells. The results showed a decrease number of EdU-positive cells and was consistent with our previous hypothesis that ZBTB28 inhibited DNA replication and gave rise to cells arrested in S phase (Fig. 3c). Besides, we observed the morphological characteristics of apoptotic cells through transmission electron microscope. The Fig. 3d revealed that, in ZBTB28 over-expression cells, increased chromatin condensation and formation of apoptotic bodies led to the weakness of inter-cellular contact and cell integrity. In addition, the AO/EB staining indicated that ZBTB28 induced near 2-fold higher number of apoptotic cells compared with the control cells (Fig. 3e). In accordance with AO/EB results, flow cytometry analysis also showed that ZBTB28 increased the proportion of both early and late apoptotic cells (Annexin V+ PI) in CaSki and HeLa (Fig. 3f). As a result, ZBTB28 could induce cell cycle arrest and increase cell apoptosis to inhibit cervical cancer.

### ZBTB28 inhibits biological functions of cervical cancer in vivo

**Fig. 4:**
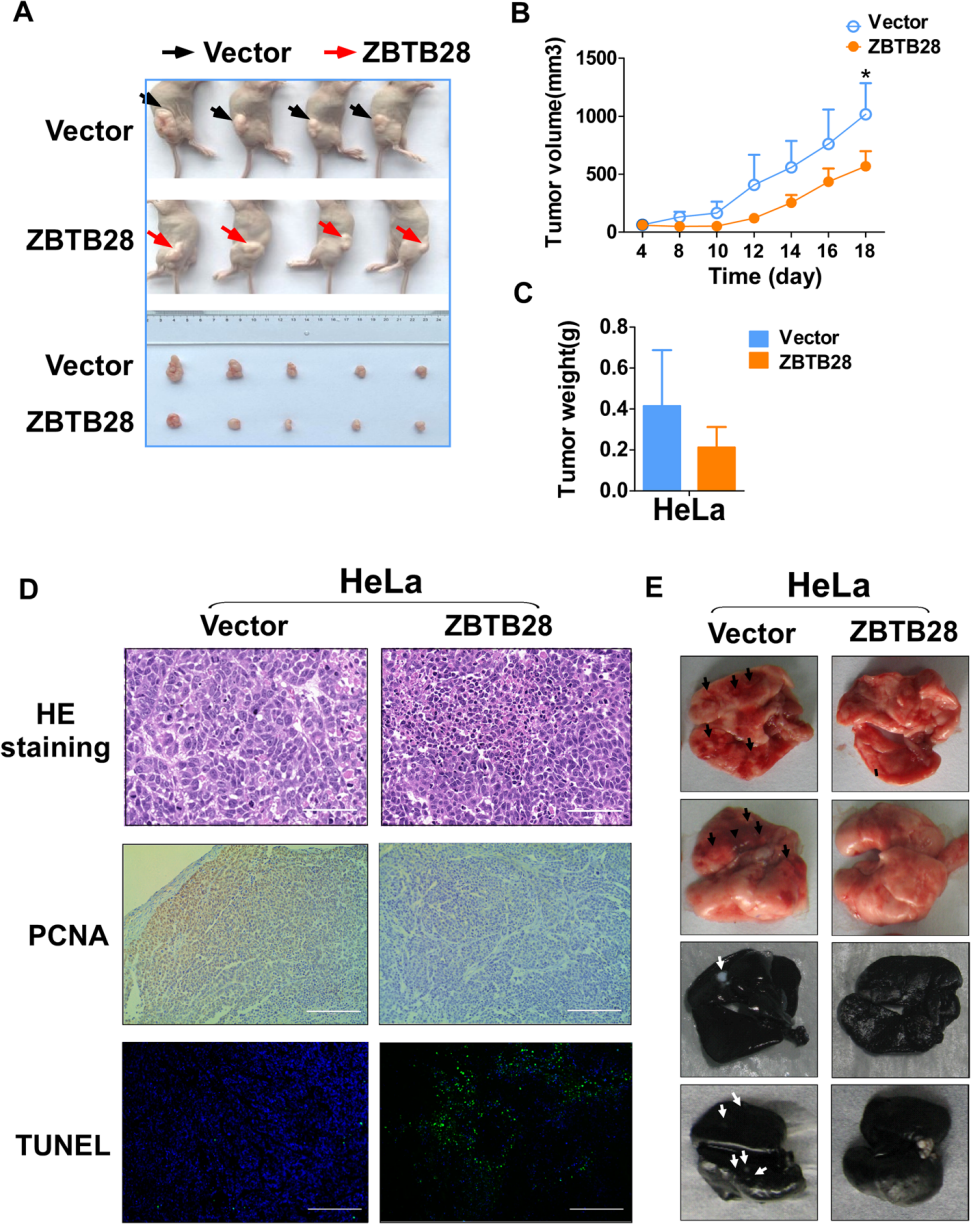
ZBTB28 suppressed tumorigenicity in vivo. **a** Representative pictures showing tumor growth 18 days after subcutaneously implanted cervical cancer cells. **b** Tumors’ volume was measured on day 4, 8, 10, 12, 14, 16, 18. **c** The weight of vector-group and ZBTB28-group tumors were measured respectively (*n* = 5). **d** Representative images of HE staining, PCNA expression. Apoptosis was assessed by TUNEL assays in xenografts. **e** HeLa cells were intravenously injected into BALB/c mice to induce lung metastasis. Lung metastatic loci were counted on day 15. All statistical data were shown as mean ± SEM. *p < 0. 05

Furthermore, we evaluated whether ZBTB28 could suppress the growth and metastasis of cervical cancer cells in vivo using nude mice model (Fig. 4a). Without significant differences in mean body weight between the two groups (data not shown), the average volume and weight of tumors in the ZBTB28 group were lower than those in the control group (Fig. 4b, c). Then we performed histological analysis (HE, PCNA and TUNEL staining) to further assess the anti-tumor ability of ZBTB28 in vivo. As indicated by HE staining, the morphological characteristics of ZBTB28 xenografts were more homogeneous than vector xenografts. Compared with higher proliferateindex in the control group, the ectopic expression of ZBTB28 inhibited proliferation of cervical cancer xenografts were demonstrated by immunohistochemical staining with a monoclonal antibody against PCNA. At the same time, TdT-mediated dUTP nick end labeling (TUNEL) staining confirmed that ZBTB28 promoted the apoptosis of cervical cancer (Fig. 4d). Furthermore, we established an artificial lung metastasis model, and there was no significant lung metastatic focus was found in the ZBTB28 group (Fig. 4e). All the results indicated that ZBTB28 could act as a tumor suppressor to inhibit subcutaneous tumor growth and pulmonary metastatic foci formation in vivo.

### ZBTB28 induced autophagy in cervical cancer cells

**Fig. 5:**
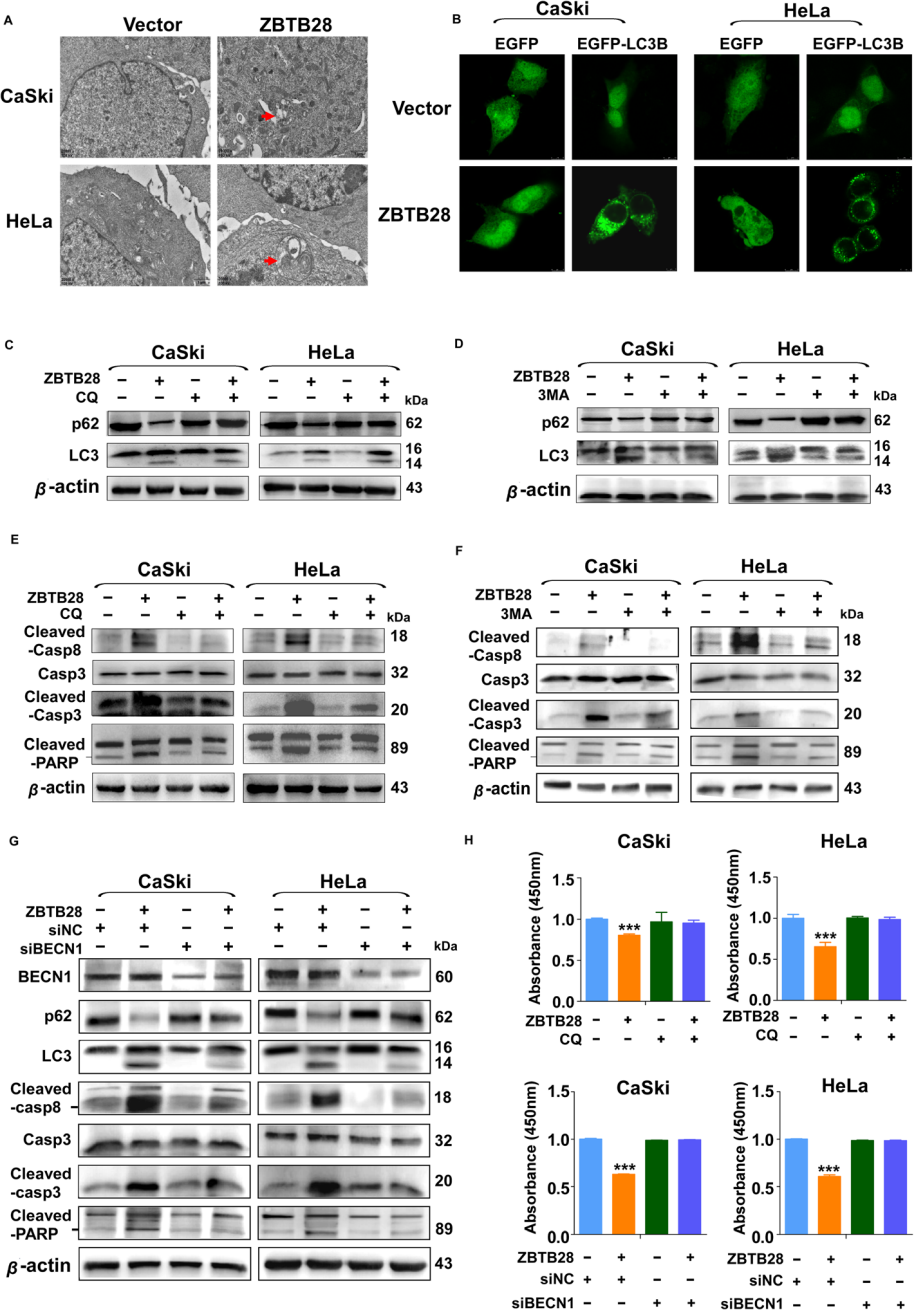
Blocking ZBTB28-induced autophagy attenuated apoptosis in cervical cancer cells. **a** The data were shown as representative TEM images of cervical cancer cells which transfected with pcDNA-3.1 or pcDNA-ZBTB28. Arrows pointed the characteristic of autophagosomes. (20,000x, Scale bar: 1 μm). **b** Stable transfected cells were transient transfected with EGFP or EGFP-LC3B. Photographs were taken under a confocal microscopy. **c**, **d** Stable transfected cells were treated with CQ (20 μM) or 3MA (10 mM) for 24 h. p62, LC3 were detected by western blot. β-actin was used as negative control. **e**, **f** Stable transfected cells were treated with CQ (20 μM) or 3MA (10 mM) for 24 h. Casp8, Casp3, and PARP were detected by western blot. β-actin was used as negative control. **g** Forty eight after transfection with siBECN1 or control siRNA, the expression of BECN1, p62, LC3, Cleaved-casp8, Casp3, Cleaved-casp3 and Cleaved-PARP were evaluated by western blot. β-actin was used as negative control. **h** Stable transfected CaSki and HeLa cells were treated with CQ (1 μM) or re-transfected with siBECN1 and control siRNA. Seventy two hours after treatment, cell viability was assessed by CCK8 assay. *p < 0. 05, **p < 0. 01, ***p < 0. 001

In our research, electron microscopic photograph showed that ZBTB28 induced the formation of double-membrane structures (the autophagosome) in cervical cancer cells (Fig. 5a). Thus, we examined whether ZBTB28 could induce autophagy in cervical cancer. During the autophagy, MAP 1LC3B/LC3B, which is scattered distributed in non-autophagy cells, experienced processing and amalgamated into puncta in cytoplasm [31]. By transient transfecting GFP-LC3B plasmid, we assessed the formation of GFP-LC3B puncta in ZBTB28 over-expression cervical cancer cells. Indeed, ZBTB28 increased the formation of LC3B puncta (Fig. 5b). This phenomenon implied that ZBTB28 enriched the formation of autophagosome in CaSki and HeLa cells. Even though the number of puncta LC3B in each cell is considered as an accurate indicator of the quantity of autophagosome, the accumulation of autophagosomes does not always represent the induction of autophagy [32]. Thus, the measurement of autophagic flux is essential to confirm that ZBTB28 induces autophagy rather than autophagosome mature hindrance. It’s generally accepted that the conversion of LC3I to LC3II concomitant with the consumption of SQSTM1/p62 is a hallmark of the increase of autophagy [33]. Interestingly, we found that LC3I have converted to LC3II and p62 was reduced after the over-expression of ZBTB28 in cervical cancer cells. To further confirm the changes of LC3 and p62 have resulted from the increase of autophagy flux, we used autophagy inhibitors chloroquine (CQ) and 3-methyladenine (3MA) to block autophagy at different stages in the turnover assay [32]. Treatment of CQ overcame the reduction of p62 induced by ZBTB28 compared with control cells. However, CQ which worked by blocking autophagosome-lysosome fusion in the later stage of autophagy had no significant effect on LC3 turnover (Fig. 5c) [34]. Thus, we used 3MA, an early-stage autophagy inhibitor inhibiting autophagosome membrane formation, to reconfirm the effect of ZBTB28-induced autophagy [35]. As expected, ZBTB28 induced the degradation of p62 and the conversion of LC3I to LC3II was restricted by 3MA (Fig. 5d). Collectively, the data clearly indicated that the pro-autophagic effect was exerted by the over-expression of ZBTB28 in cervical cancer cells.

### Autophagy is involved in ZBTB28-induced cell apoptosis

Several researches have suggested that activation of apoptosis is a common event in cancer cells in response to autophagy induction [21, 23, 25]. Based on these findings, we investigated whether there were causal links between ZBTB28-induced autophagy and apoptosis. Firstly, we chose autophagy inhibitor CQ and 3MA to block ZBTB28-induced autophagy and then examined the apoptotic effect of ZBTB28 on CaSki and HeLa cells. As expected, treatment with CQ or 3MA prevented the increase of cleaved-caspase 8, cleaved-caspase 3 and cleaved-PARP caused by ZBTB28 (Fig. 5e, f). Hence, we hypothesized that ZBTB28-induced cell apoptosis might relate to the activation of autophagy. In order to provide more evidences for the specific implication of autophagy in ZBTB28-induced apoptosis, we used siRNA targeting *BECN1* to disturb the nucleation of autophagosomes. Knockdown of BECN1 reduced ZBTB28-induced autophagy which was characterized as the inhibition of LC3 conversion and the reduction of p62 degradation. Meanwhile, downregulation of BECN1 decreased the cleavage fragment of caspase 8, caspase 3 and PARP in ZBTB28 overexpression cells (Fig. 5g). Moreover, ZBTB28-induced growth inhibition was reversed after abrogation of autophagy by silencing BECN1, and the treatment of CQ at the concentration that has no influence on cell viability attenuated the death of cervical cancer cells induced by ZBTB28 (Fig. 5h). In a word, all the data indicated that there might exist an intrinsic relationship between autophagy and apoptosis induced by ZBTB28 which needed further explore to the underlying molecular mechanism.

### ZBTB28 mediated autophagy though degradation of Bcl-XL and reduction of the Bcl-XL–BECN1 complex

**Fig. 6:**
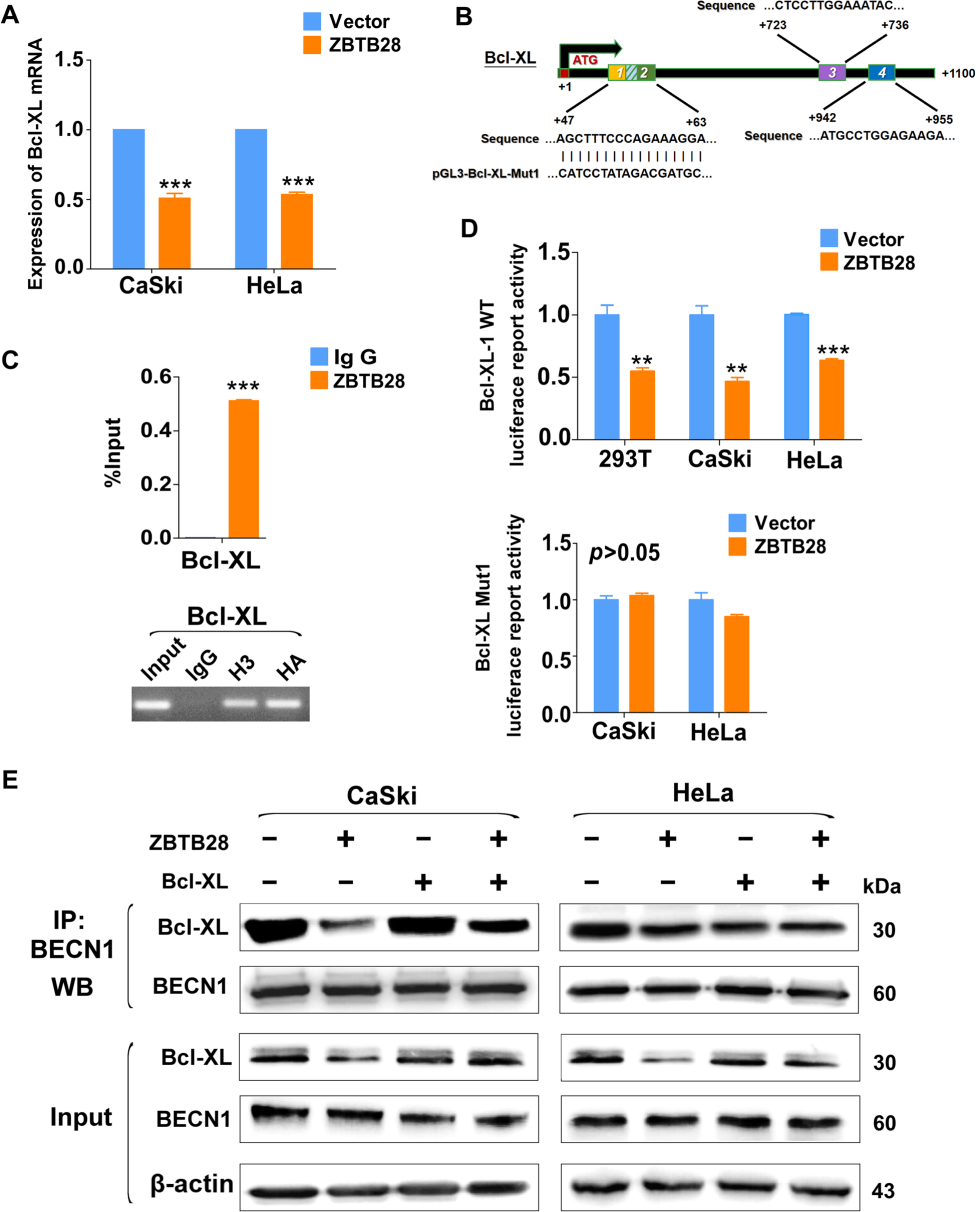
Bcl-XL plays a key role in ZBTB28-induced autophagy. **a** Expression of Bcl-XL in stable transfected cervical cancer cells. **b** Structure of wild-type and mutant Bcl-XL reporter plasmid. **c** % input of Bcl-XL DNA by HA-tag antibody were tested by ChIP-PCR. Then products of ChIP-PCR were used for electrophoresis. **d** Promoter luciferase activity of wild-type and mutant Bcl-XL in cervical cancer cells. **e** CaSki and HeLa cells were transfected with Bcl-XL. Sixty hours after transfection, the indicated proteins were detected by western blot after co-immunoprecipitation with BECN1 antibody. *p < 0. 05, **p < 0. 01, ***p < 0. 001

It had been reported that the autophagy of HeLa cells were dependent on BECN1 [36]. However, our results showed that over-expression of ZBTB28 had little effect on the expression of *BECN1*, and *siBECN1* could disturb nucleation of autophagosomes (Fig. 5g, Fig. S2A). This phenomenon attracted us to analysis the potentially mechanism underlying it. It is well-documented that Bcl-2 family proteins (BCL2, Bcl-XL and MCL1) could regulate autophagy by displacing BECN1 from the BECN1-Vps34 complex [20]. Thus qRT-PCR were performed to verify whether ZBTB28 could regulate BCL-2 family genes. The results revealed that the expression of *Bcl-XL* was downregulated by ZBTB28 while there was no influence of *BCL2* and *MCL1* (Fig. 6a, Fig. S2B, C). Further, we used online database (http://jaspar.genereg.net/) to find four predicted ZBTB28 transcription factor-binding sites (TFBSs, from + 47 to + 60, + 50 to + 63, + 723 to + 736 and + 942 to + 955) in *Bcl-XL* promoter region (Fig. 6b). Moreover, the ChIP assay confirmed that ZBTB28 was enriched at the predicted region of *Bcl-XL* promoter, while a control IgG antibody showed no significant enrichment over the entire surveyed region (Fig. 6c). Then the promoter region of *Bcl-XL* which containing different predicted TFBSs of ZBTB28 were cloned into the pGL3-Basic reporter vector, respectively. After mutation of the predicted site, the corresponding *Bcl-XL* mutant reporter plasmid was constructed. Luciferase reporter assay showed that ZBTB28 down-regulated promoter activity of *Bcl-XL* wild-type #1 but has no effect on wile-type #2 or mutant of *Bcl-XL*. (Fig. 6d, Fig. S2D). These results revealed that ZBTB28 reduced the expression of *Bcl-XL* by inhibiting its promoter activity.

Because the dissociation of Bcl-XL-BECN1 complex could trigger autophagy, we examined whether the ZBTB28-induced reduction of Bcl-XL could break the complex containing Bcl-XL and BECN1 by immunoprecipitation. The interaction between endogenous BECN1 and Bcl-XL was detected by using antibody against BECN1. Notably, as shown in Fig. 6e, we verified that BECN1 was constitutively bound to Bcl-XL and downregulated by ZBTB28. Further restoration of Bcl-XL cancelled the decrease of BECN1–Bcl-XL complex induced by ZBTB28 (Fig. 6e). Taken together, these results suggested that ZBTB28 activated autophagy by alleviating the suppression of Bcl-XL on BECN1.

### Upregulation of FIP200 promoted ZBTB28-induced autophagy to mediate cell apoptosis

**Fig. 7:**
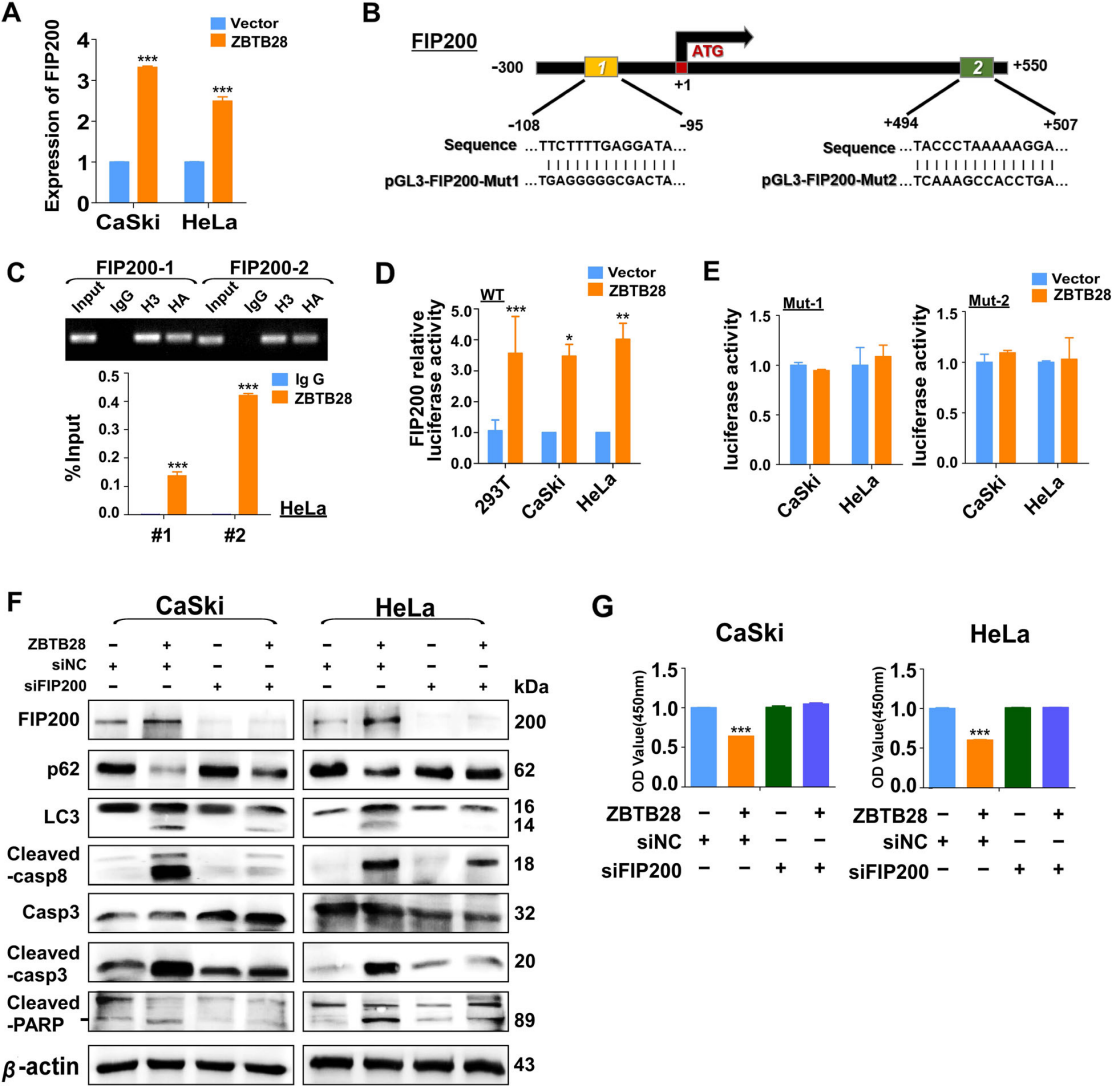
ZBTB28 upregulated FIP200 to induce autophagy and apoptosis. **a** qRT-PCR results of FIP200 mRNA expression in stable transfected cervical cancer cells. **b** Structure of wild-type and mutant FIP200 reporter plasmid. **c** % Input of FIP200 DNA by HA-tag antibody were tested by ChIP-PCR. Then products of ChIP-PCR were used for electrophoresis. **d** The effect of ZBTB28 on FIP200 promoter activity was detected by dual luciferase reporter system. **e** CaSki and HeLa cells were transfected with pcDNA3.1 or ZBTB28, then tested promoter luciferase activity of mutant FIP200 in live cells. **f** Forty eight hours after transfection with siFIP200 or control siRNA, the expression of FIP200, p62, LC3, Cleaved-casp8, Casp3, Cleaved-casp3 and Cleaved-PARP were evaluated by western blot. β-actin was used as negative control. **g** Stable transfected CaSki and HeLa cells were re-transfected with siFIP200 or control siRNA. Seventy two hours after transfection, cell viability was assessed by CCK8 assay. *p < 0. 05, **p < 0. 01, ***p < 0. 001

Our present results showed that both pharmacological inhibitors of autophagy and genetic knockdown of *BECN1* could inhibit apoptosis and concomitant with caspase inactivation. This indicated that ZBTB28-induced autophagy might trigger the apoptotic cascade. Therefore, we investigated whether ZBTB28 could interact with autophagy-related genes (*ATGs*) at transcription level with a direct effect on inducing autophagy. It was worth noticing that ZBTB28 up-regulated the expression level of endogenous *FIP200* and *ATG16L2* in both CaSki and HeLa cells while there were no significant impacts on other *ATGs* (Fig. 7a, Fig. S3A, B). Likewise, the open-access database JASPAR was used to search potential ZBTB28 TFBSs on *FIP200* and *ATG16L2* promoter. Two putative binding sites on *FIP200* promoter (positions: - 108 to - 95 and + 494 to + 507) and three sites on *ATG16L2* promoter (positions: - 991 to - 978, − 934 to - 921 and - 541 to - 533) were obtained from the database (Fig. 7b, Fig. S3C). We then conducted the ChIP assay to confirm these binding sites. ZBTB28-binding consensus sequence in *FIP200* or *ATG16L2* promoter region was examined with HA-tag antibody. The results showed that exogenous ZBTB28 expression was enriched in the predicted region which was compared with IgG (Fig. 7c, Fig. S3D). To further prove this result, we employed the luciferase reporter assay to monitor *FIP200* and *ATG16L2* promoter activity which was regulated by ZBTB28 in living cells. Consistent with our hypothesis, ZBTB28 could directly activate the *FIP200* and *ATG16L2* wild-type promoter activities in 293 T and cervical cancer cells, however it was incapable of activating the promoters of mutant one (Fig. 7d, e, Fig. S3E). It is well known that FIP200 is an indispensable component of the ULK1 complex which plays a key role in the initiation of autophagy [37]. But the role of ATG16L2 in autophagy is still controversial [38, 39]. Hence, in our research, siRNAs which targeting *FIP200* were used to block autophagy to check the causal link between ZBTB28-induced autophagy and apoptosis. The results showed that knockdown of FIP200 decreased the conversion of LC3I to LC3II and the degradation of p62 (Fig. 7f), which indicated the jamming of an autophagy response. More surprisingly, knockdown of FIP200 attenuated ZBTB28-induced apoptosis that manifested as the decrease in cleaved fragments of caspase8, caspase 3 and PARP (Fig. 7f). In addition, the deletion of FIP200 eliminated the growth inhibition of cervical cancer cells which induced by ZBTB28 (Fig. 7g). Collectively, the above results posited that ZBTB28-induced apoptosis was highly relied on FIP200-induced autophagy, and involved with activation of caspases.

### ZBTB28 increased the sensitivity to paclitaxel, cisplatin and 5-fluorouracil of cervical cancer cells

**Fig. 8:**
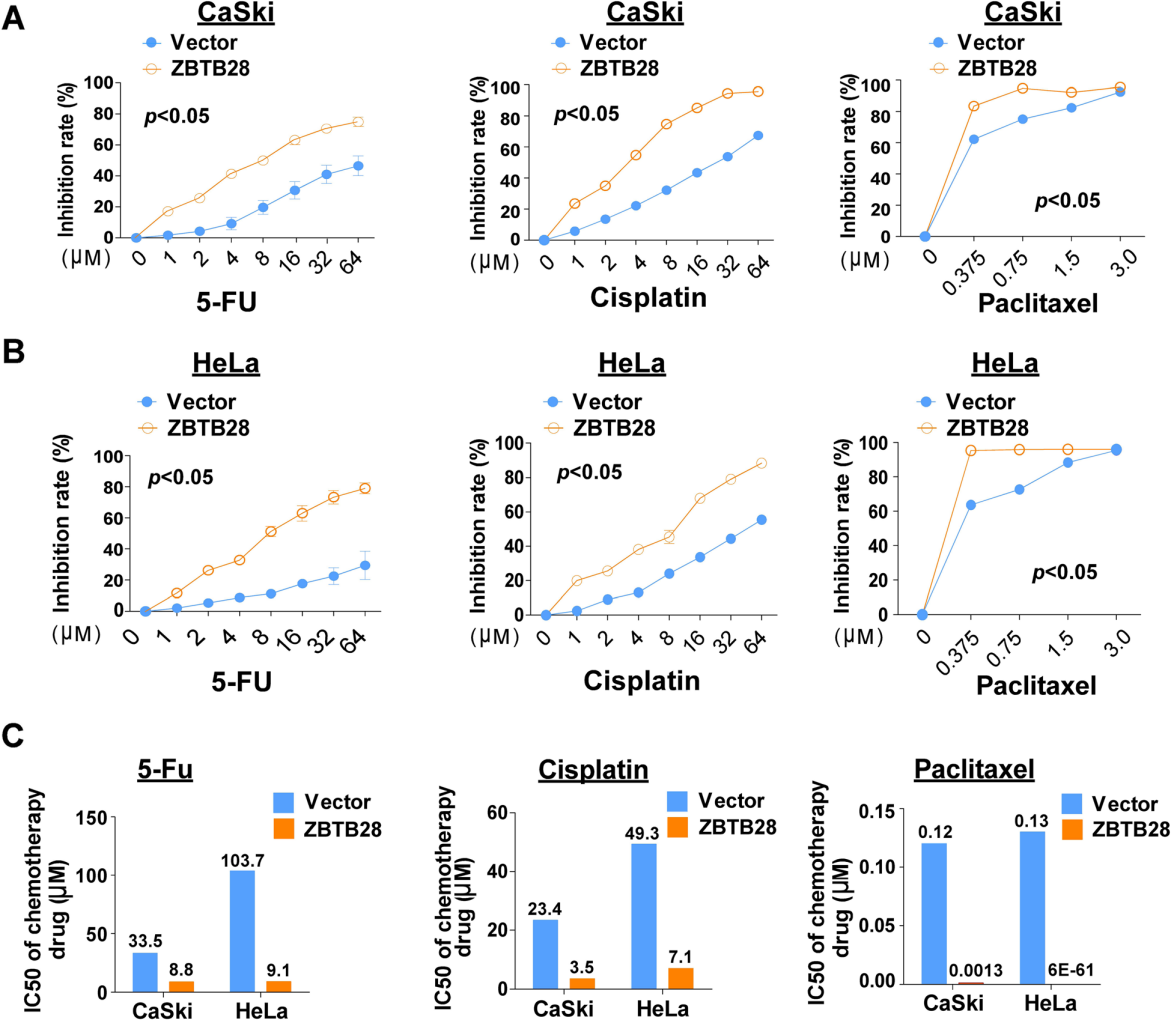
ZBTB28 sensitized the respond of cervical cancer cells to 5-FU, Cisplatin and Paclitaxel. **a**, **b** Inhibition rate of proliferation in pcDNA3.1 or ZBTB28 transfected cells treated with gradient concentration of 5-FU, Cisplatin and Paclitaxel. **c** IC50 values of 5-FU, Cisplatin and Paclitaxel for cervical cancer cells. *p < 0. 05, **p < 0. 01, ***p < 0. 001

As shown in previous results, ectopic expression of ZBTB28 induced S-phase or G0/G1-phase cell cycle arrest in cervical cancer cells. Cell cycle status could affect sensitivity of tumor cells to chemotherapeutic drugs such as Paclitaxel, Cisplatin and 5-FU. Given the combination of Paclitaxel, platinum drugs, 5-FU and radiotherapy was a safe and tolerable treatment for persistent or recurrent cervical carcinoma [40], we wondered whether ZBTB28 could influence the chemo-sensitivity of cervical tumor cells. IC50 assay was performed to detect the drug sensitivity of cells which overexpression ZBTB28. Noteworthily, the results showed an increased inhibition rate of those drugs on ZBTB28 over-expression CaSki and HeLa cell lines with a decreased IC50 of those medications (Fig. 8a-c). Specifically, ectopic expression of ZBTB28 could reduce the IC50 of 5-Fu from 33.5 to 8.8 (μM) and 103.7 to 9.1 (μM), the IC50 of Cisplatin from 23.4 to 3.5 (μM) and 49.3 to 7.1 (μM) as well as that of Paclitaxel from 0.12 to 0.0013 (μM) and 0.13 to 6E-61 (μM) in CaSki and HeLa cell lines, respectively (Fig. 8c). Therefore, our results showed that ZBTB28 could sensitize cervical cancer cells to chemotherapy reagents Paclitaxel, Cisplatin and 5-Fu. These findings opened up a new insight of the synergistic role of ZBTB28 in the chemotherapy of cervical cancer. In conclusion, ZBTB28 may be used as a potential therapeutic target in cervical cancer treatment.

## Discussion

**Fig. 9:**
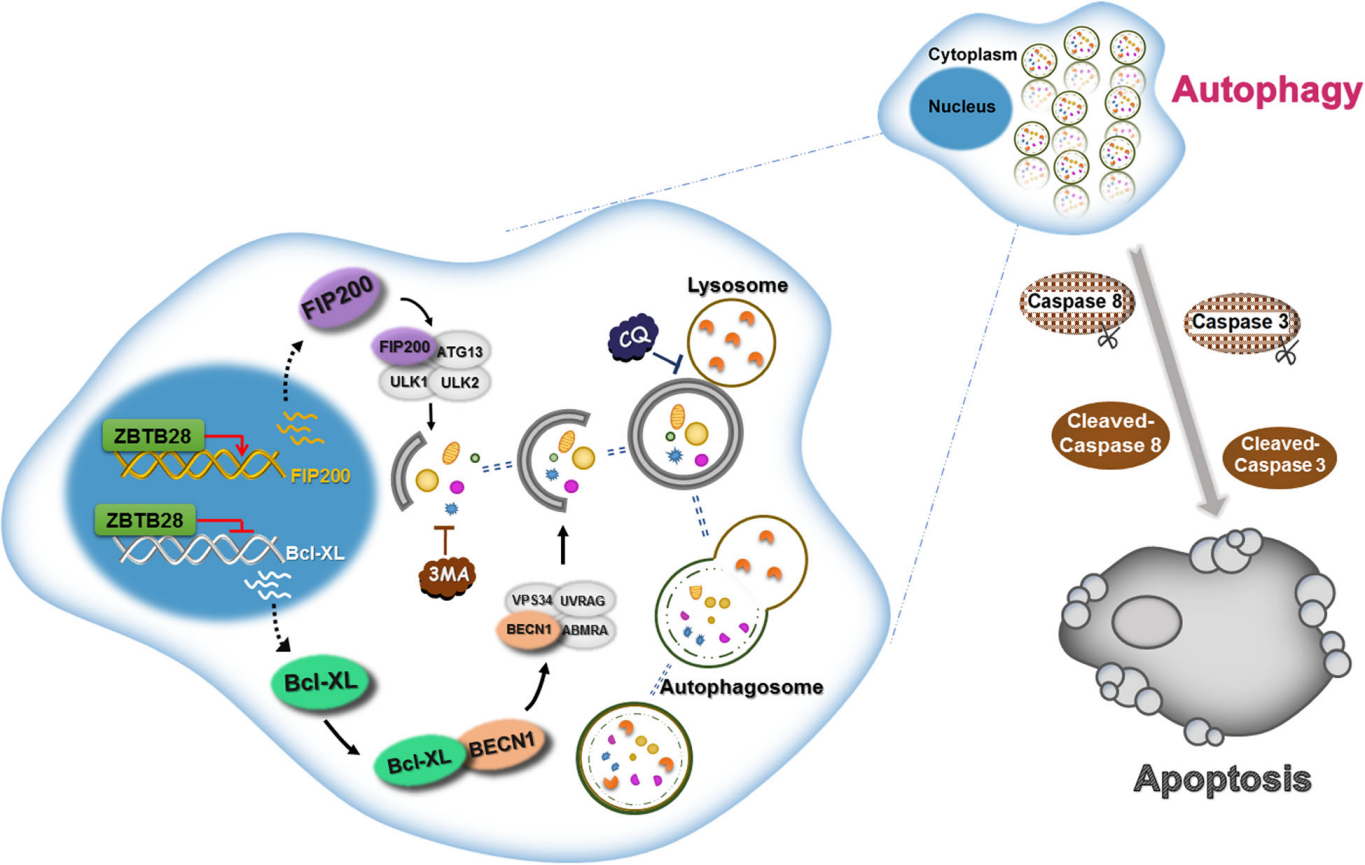
Molecular mechanism of ZBTB28 inhibiting cervical cancer

Although autophagosomes are often considered to be a tumor cell survival mechanism during stress conditions, autophagosomes can also contribute to tumor cell death [41, 42]. In this study, we assessed the transcription factor, ZBTB28, for its functional role in cervical cancer and its relationship to autophagy and apoptosis. We found ZBTB28 to inhibit cervical cancer cell malignant functions in vivo and in vitro, and demonstrated the involvement of autophagy-related apoptosis in the process (Fig. 9).

The 5-year survival rate for cervical cancer patients is greater than 90% for those who are diagnosed early and who receive timely treatment [43]. However, the survival rate drops sharply if the tumor has invaded surrounding tissues or has metastasized to other organs. Therefore, it is essential to develop new biomarkers for the early diagnosis of cervical cancer, which will improve patient quality of life. We have demonstrated DNA methylation-based silencing of *ZBTB28* to functionally participate in cervical cancer development. The silencing of tumor suppressor genes (TSGs) by DNA hypermethylation, even after human papilloma virus (HPV) clearance, triggers carcinogenesis of the cervix [44]. In this study, tumor tissue methylation of *ZBTB28* was greater than 90%, which is equivalent to the detection of high-risk *HPV* DNA (Table S1). These results suggest that the methylation status of *ZBTB28* may be a valuable marker for cervical cancer screening.

It is worth noting that other than bevacizumab, there is no U. S. Food and Drug Administration approved targeted medicine for cervical cancer [45]. Existing clinical treatment drugs are less than optimal in that they can be cytotoxic and can result in drug tolerance. As such it is necessary to explore new therapeutic targets for advanced cervical cancer patients [46]. Our drug sensitivity assays found ZBTB28 to increase the sensitivity of cervical cancer cells to the current chemo-reagents; Cisplatin, Paclitaxel, and 5-FU [47, 48]. These results suggest that ZBTB28 may improve current chemotherapies, and may become a potential therapeutic target that can guide treatment decisions for cervical cancer patients.

Further, we found autophagy to be enhanced by the re-expression of ZBTB28 in cervical cancer cells. Autophagy is a precisely regulated process that induces the formation of double-membrane vacuoles (autophagosomes) in which unnecessary or damaged organelles are delivered to lysosomes for elimination [8, 49]. We used the autophagy inhibitors, CQ and 3MA, to prevent ZBTB28-induced autophagy. Meanwhile, we found these inhibitors to attenuate the cervical cancer cell pro-apoptotic effect of ZBTB28. To exclude the possible influence of the inhibitors on other cellular processes, we knocked down BECN1 to disturb the generation of autophagosomes. Consistent with the previous results, silencing of BECN1 simultaneously blocked autophagy and apoptosis induced by ZBTB28. We did find BCEN1 to contribute to autophagy, however, which was not directly regulated by ZBTB28.

As a BH3-only protein, BECN1 interacts with Bcl-2 family members (in particular Bcl-2, Bcl-XL, and MCL1) via its BH3 domain [50]. The relative amount of BCL2 family member-BECN1 complexes within a cell impacts autophagy [20]. We found that ZBTB28 did not affect the status of *BCL2* and *MCL1*. However, degradation of Bcl-XL was a direct consequence of transcriptional and post-transcriptional regulation of ZBTB28. ZBTB28 stimulated autophagy by dissociating BECN1 from Bcl-XL. BECN1 was then liberated from its inhibition by Bcl-XL, which gave rise to autophagosomes. As a result, ZBTB28 impaired the complex of Bcl-XL-BECN1 allowing for BECN1 to enhance autophagy.

Different *ATGs* regulate different autophagy stages with the level of expression and consequent molecular interactions determinants of autophagy direction [49]. Conventional autophagy initiation of autophagosome formation usually requires two key components. One is regulated by the BECN1-Vps34 (class III PI3K) complex responding to cellular stress [51]. The other is the formation of autophagosomes dependent on the FIP200-ULK1/2-ATG13-ATG101 complex that is the upstream regulator of the autophagy pathway sensitive to cell metabolic status [31]. We have screened FIP200 as a possible target for ZBTB28 by examination of *ATG* family expression. Silencing FIP200 successfully reversed autophagy induced by ZBTB28, reducing apoptosis as well as activation of the caspase cascade. Previous studies have shown the expansion of autophagosomal membranes to be fundamental to caspase-8 self-activation [52].

During the process of autophagosome formation, the association of LC3II and p62 attracts the ATG5-ATG16L-ATG12 complex to autophagosomal membranes. Atg5 on LC3- and Atg16L-positive autophagosomal membranes associate with the Fas-associated death domain protein (FADD), an adaptor protein for caspase-8 activation. The association recruited caspase-8 to autophagosomal membranes that facilitate the development of the intracellular death-inducing signaling complex (iDISC). iDISC formation activates endogenous caspase-8 to initiate the caspase-8 cascade, resulting in autophagy-related apoptosis [52]. Further, autophagosome-lysosome fusion is involved in the maturation of CTSD (cathepsin D). CTSD exists in autolysosomes that are formed by the fusion of autophagosomes with lysosomes that are released to the aqueous component of the cytoplasm by lysosomal membrane permeabilization. M-CTSD, transported into the nucleus, triggers caspase 3 cleavage and apoptosis [53]. An in-depth understanding of the molecular basis for ZBTB28 induced autophagy-related apoptosis will provide a new theoretical framework by which to analyze the intracellular machinery of autophagy and cell death.

## Conclusions

The results of this study suggest that decreased activation of autophagy correlates with the silencing of ZBTB28 due to methylation of its promoter. ZBTB28 induces autophagy by directly promoting FIP200 and by relieving Bcl-XL suppression of BECN1. Further, apoptosis induced by ZBTB28 was found to be related to autophagy, which beneficially sensitized cervical cancer cells to chemotherapeutic reagents. Therefore, enhanced autophagic activity or a combination of autophagy agonists with chemotherapeutic reagents may be a new strategy by which to inhibit the progression of cervical cancer. In conclusion, ZBTB28 may have clinical utility as a specific biomarker for screening and diagnosis of cervical cancer and may be a target for individualized patient therapies.

## Supplementary Information

**Additional file 1: Table S1** Diagnostic efficacy of HPV and ZBTB28 methylation in cervical cancer tissues. **Table S2** List of RT-PCR primers. **Table S3** List of qRT-PCR Primers. **Table S4** List of ChIP-PCR Primers.

**Additional file 2: Figure S1**. Nucleus localization of ZBTB28. The localization of ZBTB28 and vimentin or Ecad in the nucleus of HeLa was analyzed by immunofluorescence. **Figure S2**. Regulation of ZBTB28 on Bcl-2 family proteins. (A) The expression of BECN1 was analyzed. (B, C) The expression of BCL2 and MCL1 was analyzed by qRT-PCR. (D) Promoter luciferase activity of Bcl-XL in cervical cancer cells. **Figure S3**. ZBTB28 regulated ATG16L2 in cervical cancer cells. (A, B) qRT-PCR results of ATGs mRNA expression in stable transfected cervical cancer cells. (C) Structure of wild-type ATG16L2 reporter plasmid. (D) % input of ATG16L2 DNA by HA-tag antibody were detected by ChIP-PCR. (E) Use dual luciferase reporter system to detect the effect of ZBTB28 on ATG16L2 promoter activity in 293T, CaSki and HeLa cells.

## Data Availability

The datasets used and/or analysed during the current study are available from the corresponding author on reasonable request.

## Abbreviations

3MA: 3-methyladenine
5-FU: 5-fluorouracil
5-Aza: 5-aza-2′-deoxycytidine
AO/EB: Acridine orange/ethidium bromide
ATGs: Autophagy-related genes
ATG5: Autophagy gene 5
BECN1: Beclin1
ChIP: Chromatin immunoprecipitation
CQ: Chloroquine
EdU: 5-ethynyl-2′-deoxyuridine
EMT: Epithelial mesenchymal transition
FIP200: FAK Family Kinase-Interacting Protein Of 200 KDa
IC50: Half maximal inhibitory concentration
LC3I: The nonlipidated form of LC3B
LC3II: The lipidated form of LC3B
MAP 1 LC3B/LC3B: Microtubule-associated protein 1 light chain 3 beta
MSP: Methylation-specific PCR
p62: SQSTM1/p62
ZBTB28: Zinc-finger and BTB/POZ (Poxvirus and Zinc-finger) domain-containing family protein 28
