# Supplementary material for: ZBTB28 induces autophagy by regulation of FIP200 and Bcl-XL facilitating cervical cancer cell apoptosis
Source: J Exp Clin Cancer Res. 2021 Apr 30;40:150. doi: 10.1186/s13046-021-01948-0 (PMC8086320; doi:10.1186/s13046-021-01948-0)
Supplement: Supplementary file 1 — Additional file 1: Table S1 Diagnostic efficacy of HPV and ZBTB28 methylation in cervical cancer tissues. Table S2 List of RT-PCR primers. Table S3 List of qRT-PCR Primers. Table S4 List of ChIP-PCR Primers. [file 13046_2021_1948_MOESM1_ESM.pdf]

Table.S1 Diagnostic efficacy of HPV and ZBTB28 methylation in cervical cancer tissues

	HPV	ZBTB28	P value
Sensibility	32/48 (66.7%)	45/48 (93.8%)	0.254
Specificity	6/20 (30.0%)	17/20 (85.0%)	0.202
AUC	0.517	0.894	<0.001

Table.S2 List of RT-PCR primers

PCR Primer	Sequence (5'-3')	Product size(bp)
ZBTB28F	CTACGTCCGCGAGTTCACTC	170bp
ZBTB28R	CCCGGAAAATTGAATAGAAG	
VIMF	TGCCAACCTTTACAGACCTA	390bp
VIMR	CTCATCTCCCTCCTCACTCA	
EcadF	CCTCCGTTTCTGGAATCCAA	282bp
EcadR	GTTCTCTATCCAGAGGCTCT	
NcadF	CAGGTTTGGAATGGGACAGT	480bp
NcadR	TCCAGTAGGATCTCCGCCAC	
HPV16F	TTAGGCAGCACTTGGCCAACCA	207bp
HPV16R	TAATCCGTCCTTTGTGTGAGCT	
HPV18F	CCGAGCACGACAGGAACGACT	173bp
HPV18R	TCGTTTTCTTCCTCTGAGTCGCTT	
β-actinF	TCCTGTGGCATCCACGAAACT	315bp
β-actinR	GAAGCATTTGCGGTGGACGAT	

Table.S3 List of qRT-PCR Primers

PCR Primer		Sequence (5'-3')	Product size (bp)	Annealing temperature (℃)
	ULK1F	AGCACGATTTGGAGGTCGC	118bp	60
	ULK1R	GCCACGATGTTTTCATGTTTCA		
	ULK2F	GTGGTATTCGCATCAAAATAGCG	145bp	60
	ULK2R	CACAAGTCAGCCTTAGCATCATA		
	ATG2BF	GGACGGTTAATTGGTAGGTTGG	222bp	60
	ATG2BR	CTGCATGGGTCGATTTTTCCT		
	ATG3F	GATGGCGGATGGGTAGATACA	125bp	60
	ATG3R	TCTTCACATAGTGCTGAGCAATC		
	ATG4BF	GGTGTGGACAGATGATCTTTGC	172bp	60
	ATG4BR	CCAACTCCCATTTGCGCTATC		
	ATG5F	AGAAGCTGTTTCGTCCTGTGG	152bp	60
	ATG5R	AGGTGTTTCCAACATTGGCTC		
	ATG7F	CTGCCAGCTCGCTTAACATTG	216bp	60
	ATG7R	CTTGTTGAGGAGTACAGGGTTTT		
	ATG9AF	CCAGAACTACATGGTGGCACT	131bp	60
	ATG9AR	GTCCCCAGAAGAGGATCAGC		
	ATG10F	CCCTTGGATGATTGTGAAGTGA	195bp	60
	ATG10R	CTGTAGCAGTCGCATCTTATAGC		
	ATG12F	TAGAGCGAACACGAACCATCC	153bp	60
	ATG12R	CACTGCCAAAACACTCATAGAGA		
qRT-PCR	ATG13F	TTGCTATAACTAGGGTGACACCA	137bp	60
	ATG13R	CCCAACACGAACTGTCTGGA		
	ATG14F	GCGCCAAATGCGTTCAGAG	91bp	60
	ATG14R	AGTCGGCTTAACCTTTCCTTCT		
	ATG16L1F	AACGCTGTGCAGTTCAGTCC	177bp	60
	ATG16L1R	AGCTGCTAAGAGGTAAGATCCA		
	ATG16L2F	TTAGCAGCAACTTACAACCAGG	226bp	60
	ATG16L2R	ACACCACGTCATTACAGTAGGA		
	FIP200F	GAAAGAGCTTGCTCAGGGATT	107bp	60
	FIP200R	TCATCAACTGATTTGCGTGACT		
	WIPI2F	CCATCGTCAGCCTTAAAGCAC	136bp	60
	WIPI2R	TCCAGGCATACTATCAGCCTC		
	WIPI3F	CTCCTGCCGTGTAACCCTC	250bp	60
	WIPI3R	CCCAGATCATTACTTTGTTGGGA		
	WIPI4F	GAGAAGCAACTGCTAGTGTTCC	146bp	60
	WIPI4R	GGCTGGTTTAGAGACACACAG		
	ATG101F	TTCATCGACTTCACTTATGTGCG	177bp	60
	ATG101R	GATGCACTCGTCTGAGAATGG		

	EPG5F	AAGGCCAGCCGGACTAAAAC	161bp	60
	EPG5R	TGGGAATCAGTTACCACCTTCA		
	AMBRA1F	TGGGGAGGTTAGGATTTGGGA	98bp	60
	AMBRA1R	GAGCCGTAGGGTGGAAAGC		
	NBR1F	AGGAGCAAAACGACTAGCTGC	96bp	60
	NBR1R	TCTGGGGTCTTCATGTCTGAT		
	VMP1F	GACCAGAGACGTGTAGCAATG	212bp	60
	VMP1R	ACAATGCTTTGACGATGCCATAA		
	NANOGF	ATGAGTGTGGATCCAGCTTG	190bp	60
	NANOGR	CCTGAATAAGCAGATCCATGG		
	OCT4F	AGCGATCAAGCAGCGACTAT	118bp	60
	OCT4R	TAGCCTGGGGTACCAAAATG		
	KLF4F	TCCCATCTTTCTCCACGTTC	262bp	60
	KLF4R	TCCAGGAGATCGTTGAACTC		
	ABCG2F	AACCTGGTCTCAACGCCAT	188bp	60
	ABCG2R	CAGAGTGCCCATCACAACA		
	BMI1F	GATCACTGAGCTAAATCCCC	287bp	60
	BMI1R	CAGAAGGATGAGCTGCATAAA		
qRT-PCR	MYCF	GGAGGCTATTCTGCCCATTT	177bp	60
	MYCR	GTCGAGGTCATAGTTCCTGTTGG		
	TIP30F	GGAAGACTTCAGGATGCAGA	235bp	60
	TIP30R	GAATCCAACATCATGACCTTG		
	MAD2F	AAATCGTGGCCGAGTTCTTC	244bp	60
	MAD2R	CTTTCCAGGACCTCACCACT		
	STAT3F	CCAATGGAATCAGCTACAGC	236bp	60
	STAT3R	GCTGATAGAGAACATTCGACTC		
	CD44F	TGGACAAGTTTTGGTGGCAC	176bp	60
	CD44R	GGTGCTATTGAAAGCCTTGC		
	BECN1F	CCATGCAGGTGAGCTTCGT	215bp	60
	BECN1R	GAATCTGCGAGAGACACCATC		
	Bcl-XLF	GAGCTGGTGGTTGACTTTCTC	119bp	60
	Bcl-XLR	TCCATCTCCGATTCAGTCCCT		
	BCL2F	ATGTGTGTGGAGAGCGTCAACC	196bp	60
	BCL2R	TGAGCAGAGTCTTCAGAGACAGC		
	MCL1F	TGCTTCGGAAACTGGACATCA	135bp	60
	MCL1R	TAGCCACAAAGGCACCAAAAG		
	β-actinF1	GTCTTCCCCTCCATCGTG	113bp	60
	β-actinR1	AGGGTGAGGATGCCTCTCTT		

Table.S4 List of ChIP-PCR Primers

**Primer**	**Sequence (5'-3')**	**Product size (bp)**	**Annealing temperature (℃)**
chip Bcl-XL F	TCAGTGAGTGAGCAGGTGTT	225bp	60
chip Bcl-XL R	ATGGGTTGCCATTGATGGCA		
chip FIP200 F1	GAGCATCACAGCACTCACAA	169bp	60
chip FIP200 R1	GCAAATAGTTATTGGGGCGC		
chip FIP200 F2	CGCACCCGACCTTCTTTTAA	146bp	60
chip FIP200 R2	CGGACGAAAGCAAATGCTGT		
chip ATG16L2 F1	TTCGTGTCTCACAGTAGCCT	159bp	60
chip ATG16L2 R1	AGACGCTTCACCCCATT		
chip ATG16L2 F2	TTTTCGTGTCACGGAACCTC	170bp	60
chip ATG16L2 R2	GCAGCAGGACAGCAAGATAA		
