# Supplementary material for: ZBTB28 induces autophagy by regulation of FIP200 and Bcl-XL facilitating cervical cancer cell apoptosis
Source: J Exp Clin Cancer Res. 2021 Apr 30;40:150. doi: 10.1186/s13046-021-01948-0 (PMC8086320; doi:10.1186/s13046-021-01948-0)
Supplement: Supplementary file 2 — Additional file 2: Figure S1. Nucleus localization of ZBTB28. The localization of ZBTB28 and vimentin or Ecad in the nucleus of HeLa was analyzed by immunofluorescence. Figure S2. Regulation of ZBTB28 on Bcl-2 family proteins. (A) The expression of BECN1 was analyzed. (B, C) The expression of BCL2 and MCL1 was analyzed by qRT-PCR. (D) Promoter luciferase activity of Bcl-XL in cervical cancer cells. Figure S3. ZBTB28 regulated ATG16L2 in cervical cancer cells. (A, B) qRT-PCR results of ATGs mRNA expression in stable transfected cervical cancer cells. (C) Structure of wild-type ATG16L2 reporter plasmid. (D) % input of ATG16L2 DNA by HA-tag antibody were detected by ChIP-PCR. (E) Use dual luciferase reporter system to detect the effect of ZBTB28 on ATG16L2 promoter activity in 293T, CaSki and HeLa cells. [file 13046_2021_1948_MOESM2_ESM.pdf]

Supplemental figures:

Figure S1. Nucleus localization of ZBTB28. The localization of ZBTB28 and vimentin or Ecad in the nucleus of HeLa was analyzed by immunofluorescence.

Figure S2. Regulation of ZBTB28 on Bcl-2 family proteins. (A) The expression of BECN1 was analyzed. (B, C) The expression of BCL2 and MCL1 was analyzed by qRT-PCR. (D) Promoter luciferase activity of Bcl-XL in cervical cancer cells.

Figure S3. ZBTB28 regulated ATG16L2 in cervical cancer cells. (A, B) qRT-PCR results of ATGs mRNA expression in stable transfected cervical cancer cells. (C) Structure of wild-type ATG16L2 reporter plasmid. (D) % input of ATG16L2 DNA by HA-tag antibody were detected by ChIP-PCR. (E) Use dual luciferase reporter system to detect the effect of ZBTB28 on ATG16L2 promoter activity in 293T, CaSki and HeLa cells.

Figure S1

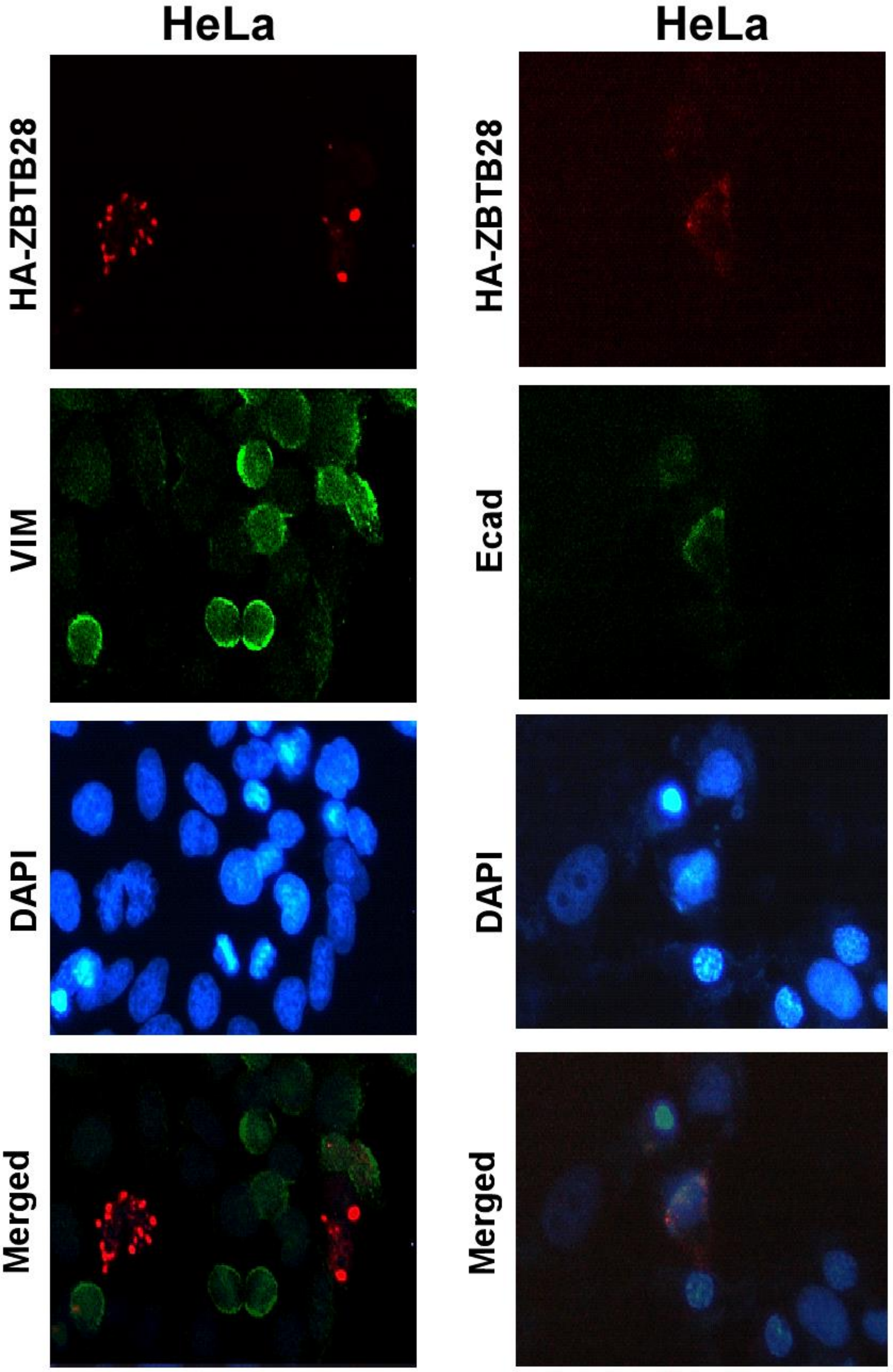
HeLa
HeLa
HA-ZBTB28
VIM
DAPI
Merged
HA-ZBTB28
Ecad
DAPI
Merged

Figure S2

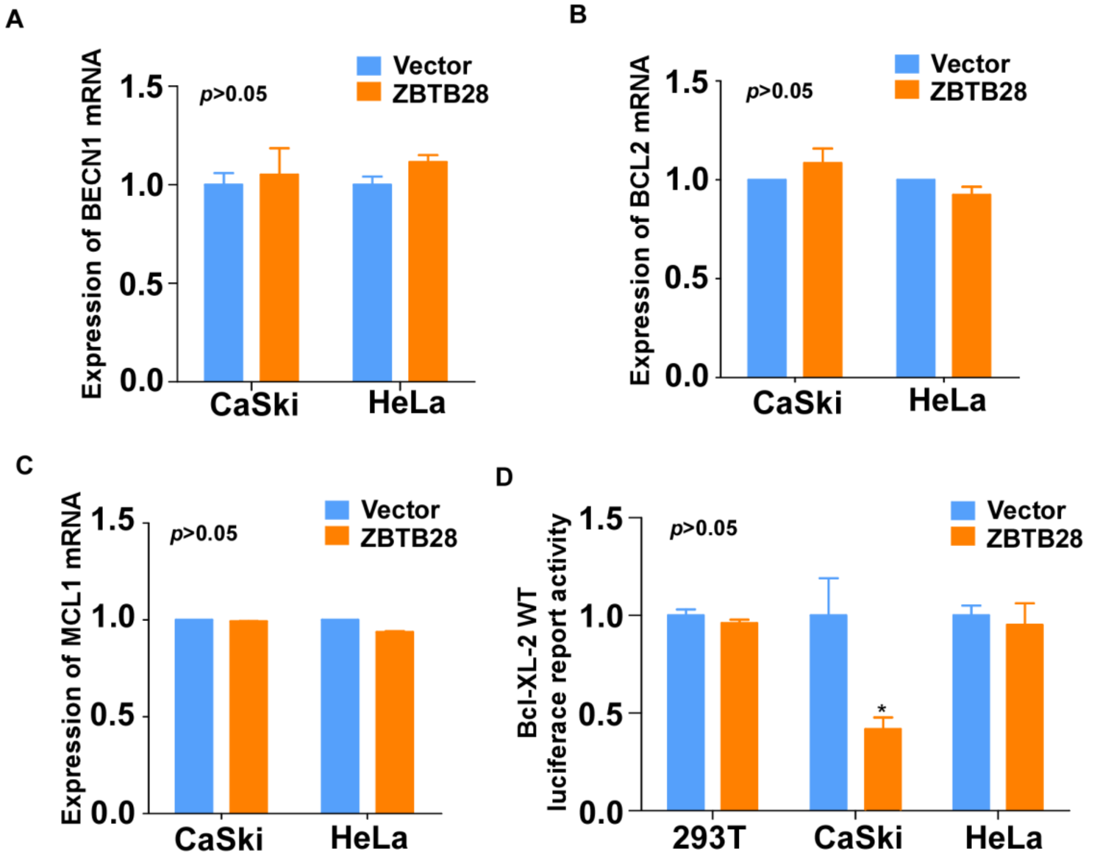
A
Expression of BECN1 mRNA
1.5
1.0
0.5
0.0
p>0.05
Vector
ZBTB28
CaSki
HeLa
B
Expression of BCL2 mRNA
1.5
1.0
0.5
0.0
p>0.05
Vector
ZBTB28
CaSki
HeLa
C
Expression of MCL1 mRNA
1.5
1.0
0.5
0.0
p>0.05
Vector
ZBTB28
CaSki
HeLa
D
Bcl-XL-2 WT
luciferace report activity
1.5
1.0
0.5
0.0
p>0.05
*
Vector
ZBTB28
293T
CaSki
HeLa

Figure S3

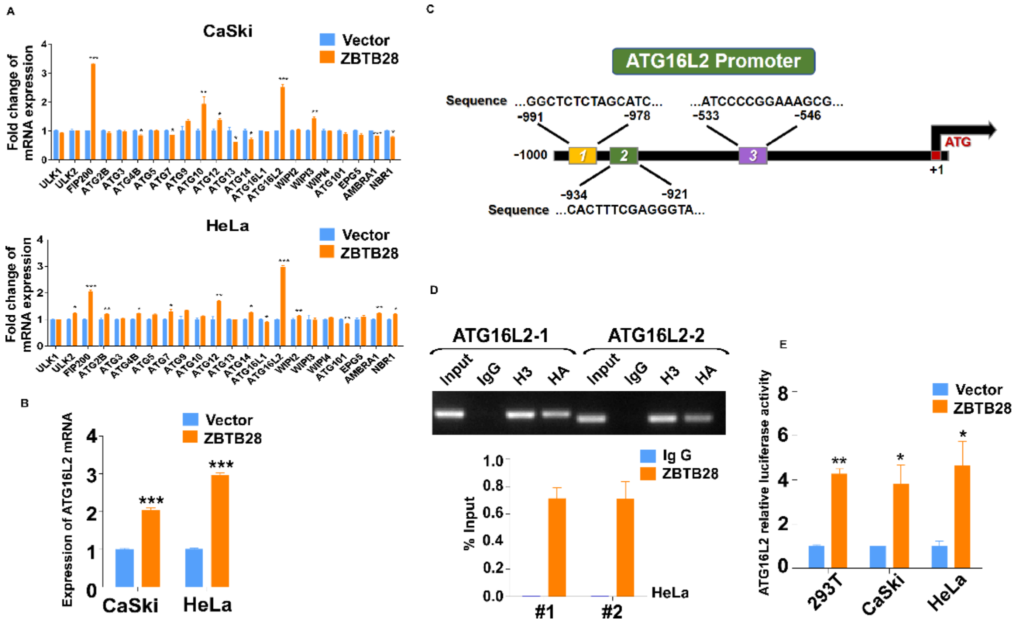
A
CaSki
Vector
ZBTB28
Fold change of mRNA expression
ULK1 ULK2 FIP200 ATG2B ATG3 ATG4B ATG5 ATG7 ATG9 ATG10 ATG12 ATG13 ATG14 ATG16L1 ATG16L2 WIPI2 WIPI3 WIPI4 ATG101 EPG5 AMBRA1 NBR1
HeLa
B
Expression of ATG16L2 mRNA
CaSki
HeLa
C
ATG16L2 Promoter
Sequence ...GGCTCTCTAGCATC...
-991
-978
...ATCCCCGGAAAGCG...
-533
-546
-1000
ATG
+1
-934
-921
Sequence ...CACTTTCGAGGGTA...
D
ATG16L2-1
ATG16L2-2
Input
IgG
H3
HA
% Input
Ig G
ZBTB28
HeLa
#1
#2
E
ATG16L2 relative luciferase activity
293T
CaSki
HeLa
